# Performance assessment of hybrid machine learning approaches for breast cancer and recurrence prediction

**DOI:** 10.1371/journal.pone.0304768

**Published:** 2024-08-01

**Authors:** Abhilash Pati, Amrutanshu Panigrahi, Manoranjan Parhi, Jayant Giri, Hong Qin, Saurav Mallik, Sambit Ranjan Pattanayak, Umang Kumar Agrawal

**Affiliations:** 1 Department of Computer Science and Engineering, Siksha ‘O’ Anusandhan (Deemed to be University), Bhubaneswar, Odisha, India; 2 Centre for Data Science, Siksha ‘O’ Anusandhan (Deemed to be University), Bhubaneswar, Odisha, India; 3 Department of Mechanical Engineering, Yeshwantrao Chavan College of Engineering, Nagpur, India; 4 Department of VLSI Microelectronics, Saveetha School of Engineering, Saveetha Institute of Medical and Technical Sciences (SIMATS), Saveetha University, Chennai, Tamil Nadu, India; 5 Department of Computer Science and Engineering, University of Tennessee at Chattanooga, Chattanooga, Tamil Nadu, United States of America; 6 Department of Environmental Health, Harvard T H Chan School of Public Health, Boston, Massachusetts, United States of America; 7 School of Computer Engineering, KIIT (Deemed to be University), Bhubaneswar, Odisha, India; North-West University Potchefstroom Campus: North-West University, SOUTH AFRICA

## Abstract

Breast cancer is a major health concern for women everywhere and a major killer of women. Malignant tumors may be distinguished from benign ones, allowing for early diagnosis of this disease. Therefore, doctors need an accurate method of diagnosing tumors as either malignant or benign. Even if therapy begins immediately after diagnosis, some cancer cells may persist in the body, increasing the risk of a recurrence. Metastasis and recurrence are the leading causes of death from breast cancer. Therefore, detecting a return of breast cancer early has become a pressing medical issue. Evaluating and contrasting various Machine Learning (ML) techniques for breast cancer and recurrence prediction is crucial to choosing the best successful method. Inaccurate forecasts are common when using datasets with a large number of attributes. This study addresses the need for effective feature selection and optimization methods by introducing Recursive Feature Elimination (RFE) and Grey Wolf Optimizer (GWO), in response to the limitations observed in existing approaches. In this research, the performance evaluation of methods is enhanced by employing the RFE and GWO, considering the Wisconsin Diagnostic Breast Cancer (WDBC) and Wisconsin Prognostic Breast Cancer (WPBC) datasets taken from the UCI-ML repository. Various preprocessing techniques are applied to raw data, including imputation, scaling, and others. In the second step, relevant feature correlations are used with RFE to narrow down candidate discriminative features. The GWO chooses the best possible combination of attributes for the most accurate result in the next step. We use seven ML classifiers in both datasets to make a binary decision. On the WDBC and WPBC datasets, several experiments have shown accuracies of 98.25% and 93.27%, precisions of 98.13% and 95.56%, sensitivities of 99.06% and 96.63%, specificities of 96.92% and 73.33%, F1-scores of 98.59% and 96.09% and AUCs of 0.982 and 0.936, respectively. The hybrid approach’s superior feature selection improved the accuracy of breast cancer performance indicators and recurrence classification.

## Introduction

The fact that it kills so many women is why breast cancer continues to be a serious public health problem worldwide. Breast cancer is the most frequent malignancy in women, with an estimated 2.3 million cases in 2020 and an estimated 685,000 deaths worldwide [1, 2]. The unchecked growth of carcinogenic, malignant tumors in the breast initiates the local spread of cancerous cells. Women of all ages and ethnicities are more at risk due to increased prevalence rates. Metastasis and recurrence (or relapse) of breast cancer are major causes of mortality. Breast cancer can recur years or even decades after it has been treated. Early detection and diagnosis of breast cancer greatly improves prognosis and survival time for people with this disease. An individual will be subjected to fewer unnecessary operations if the cancerous mass is identified. As a result, research into the most effective method of diagnosing breast cancer has to be prioritized [3, 4].

Due to its unique characteristics in the identification of variables from complicated datasets of cancer disorders, machine learning (ML) is widely employed in addressing cancer classification and model forecasting. Breast cancer diagnosis and prognosis pose significant difficulties for the medical surgeon. ML methods have greatly aided in the early diagnosis of cancer. The application built on ML methods improves the accuracy of breast cancer diagnosis and prognosis. This has steadily decreased breast cancer mortality over the past two decades [5].

Feature selection plays a crucial role in enhancing the accuracy and efficiency of predictive models, especially in the realm of breast cancer and breast cancer relapse prediction. With the increasing availability of biomedical data, identifying relevant features from a large pool of variables becomes essential to building robust and interpretable models. Feature selection methods aid in selecting a subset of informative features, contributing to the development of accurate and computationally efficient prediction models. In the context of breast cancer research, these methods are pivotal for identifying key biomarkers and risk factors that influence the progression and relapse of the disease. Additionally, Optimization techniques play a crucial role in improving the accuracy and efficiency of breast cancer and relapse prediction models. The selection of optimization techniques depends on the characteristics of the dataset, the nature of the problem, and the specific requirements of the prediction model. Combining multiple optimization strategies often leads to improved model performance and robustness. Researchers often leverage various feature selection and optimization techniques to discern meaningful patterns from diverse datasets, improving the precision of predictive models and advancing our understanding of breast cancer dynamics [6, 7].

### Research gap and motivation

Breast cancer is a disease that affects women of all races, and its incidence increases with age. Among female cancer patients, it accounts for the vast majority of fatalities. Early identification and prediction have been proposed as effective strategies for combating this aggressive cancer. Thus, ML-based recurrence prediction for breast cancer is a pressing medical issue that poses significant hurdles to the scientific community. Predicting how breast cancer will behave is crucial because it helps doctors choose the best course of action for each individual patient, leading to better outcomes. It’s also linked to better deployment of healthcare resources for such people. Both physicians and data scientists agree that elucidating the causes of breast cancer recurrence so early is a pressing area of study. Several ML algorithms and statistical approaches, which have led to better breast cancer diagnosis and prediction, have been applied in several investigations of cancer recurrence. Multiple models have been proposed in recent years to predict whether or not breast cancer will return in the next years of surgery; however, all of these models have severe drawbacks. Several attempts have been made to employ ML techniques to foretell a woman’s survival after being diagnosed with breast cancer. This is because of proactive breast cancer diagnosis and prognosis and the revolutionary treatment methods currently under development.

Breast cancer and its recurrence prediction have been the subject of extensive research, with a predominant focus on clinical perspectives. However, within the realm of optimization and feature selection methodologies, a distinct gap persists in the current state-of-the-art literature. Existing studies often employ traditional feature selection and optimization methods, such as Recursive Feature Elimination (RFE) and genetic algorithms. While these methods have demonstrated effectiveness, the landscape lacks a comprehensive exploration of hybrid models that integrate RFE, Grey Wolf Optimizer (GWO), and diverse machine learning (ML) algorithms. A critical examination of the literature reveals limited instances where these specific techniques are harmoniously combined to enhance the predictive accuracy of breast cancer recurrence models. Moreover, the existing literature predominantly highlights the medical aspects of breast cancer recurrence prediction, neglecting an in-depth exploration of the methodology employed. This study endeavors to bridge this research gap by not only providing a medical perspective but also placing a significant emphasis on the unique methodology employed. The current state-of-the-art lacks an exhaustive analysis of the potential synergies and improvements that can be achieved through the integration of RFE, GWO, and ML algorithms for feature selection, optimization, and classification in the context of breast cancer recurrence prediction. By addressing these gaps, this research aims to contribute to the refinement of methodologies for breast cancer recurrence prediction, offering a more holistic and efficient approach that extends beyond the traditional medical focus.

### Research questions

The research questions (RQs) that are being studied are as follows:

RQ1. What is the importance of hybridizing various feature selection, optimization, and classification techniques in disease prediction?
RQ2. Can we achieve 100% accuracy utilizing the hybridization of feature selection and optimization techniques along with ML classifiers on breast cancer datasets?
RQ3. Whether the proposed hybrid model can be able to detect breast cancer as well as its recurrence at its earlier stages?
RQ4. Whether this proposed hybrid approach outperformed other existing state-of-the-art models or not?

### Objective

In this paper, a hybrid ML approach is proposed based on Recursive Feature Elimination (RFE) as a feature selection technique, Grey Wolf Optimization (GWO) as an optimization technique, and seven conventional classification techniques like Naïve Bayes (NB), K-Nearest Neighbor (KNN), Logistic Regression (LR), Support Vector Machine (SVM), Multi-Layer Perceptron (MLP), Random Forest (RF) and Decision Tree (DT). The various experiments are performed on Wisconsin Diagnostic Breast Cancer (WDBC) and Wisconsin Prognostic Breast Cancer (WPBC) datasets sourced from the open access warehouse of the University of California, Irvine—Machine Learning (UCI-ML), considering ten evaluative measures.

### Contributions

The contribution of this study has been sketched up as follows:

- Developed the hybrid ML-based approach employing feature selection, optimization, and classification techniques for breast cancer and its recurrence prediction obtaining enhanced performance outcomes;
- Implemented iterative RFE and GWO cycles allowing for dynamic feature selection, adapting to changes in data patterns and enhancing the ability to capture the temporal dynamics of breast cancer progression and relapse;
- Integrated RFE with GWO for feature selection improving the interpretability and efficiency of the classification model;
- Compared and contrasted the proposed hybrid ML-based approach with some of the similar state-of-the-art works showing the novelty and significance of the study;

### Paper structure

The study of this proposed work has been organized as follows: Section 2 discusses the research work being conducted in this field with a summary table. Section 3 represents the employment of the proposed dataset along with various techniques adopted in this work. Section 4 covers the study’s architectural facet, including the proposed work’s design, flow chart, block diagrams, and working principle. Section 5 describes the productive investigation of the proposed work, in contrast with the related results considered in this study. Section 6 winds up the study with an achievable extension to the proposed work.

## Related works

Gupta and Gupta [8] introduced a comparative study of breast cancer diagnosis models considering ML approaches, including MLP, C4.5 DT, SVM, and KNN on WBCD and WDBC datasets. They resulted in 98.12% accuracy, 100% precision, and 97.85% recall. Jafarpisheh et al. [9] introduced breast cancer relapse prognosis by classic and modern structures models considering deep neural network (DNN), Rough neural network (RNN), MLP, and SVM approaches on breast cancer relapse dataset, resulting in 94.53% accuracy. Ferroni et al. [10] developed an ML-based decision support system (DSS), combined with random optimization (RO), to extract prognostic information from routinely collected demographic, clinical, and biochemical data of breast cancer patients. on the SEER dataset and resulted in 86.0% accuracy, 69.8% f-measure, 67.1% sensitivity, 88.4% specificity, 0.822 AUC. Bayrak et al. [11] developed a breast cancer diagnosis model considering ML approaches, including SVM, MLP, and Sequential Minimal Optimization (SMO) on WBCD datasets. They resulted in 96.99% accuracy, 97% precision, 97% recall, and 0.968 AUC. Naveen et al. [12] introduced an efficient breast cancer prediction model considering ML approaches, including DT, SVM, MLP, KNN, LR, and RF, on the Coimbra breast cancer dataset, which was taken from UCI and resulted in 100% accuracy, 100% precision, 100% recall, and 100% F1-score.

Zeng et al. [13] introduced identifying breast cancer distant recurrences model from electronic health records (EHRs) considering SVM, MLP, and CNN approaches on electronic health records and resulted in 81.47% precision, 77.82% recall, 79.42% F1-score, and 0.9489 AUC. Omondiagbe et al. [14] developed a computer-aided detection (CAD) system for breast cancer diagnosis considering SVM, ANN, NB, linear discriminant analysis (LDA) approaches on the WDBC dataset and resulted in 98.82% accuracy, 98.41% sensitivity, 99.07% specificity, and 0.9994 AUC. Shravya et al. [15] proposed a breast cancer prediction model considering ML approaches, including LR, SVM, and KNN, along with a dimensionality reduction technique, i.e., principal component analysis (PCA), on a UCI-resourced dataset. They resulted in 92.78% accuracy, 96.55% precision, 91.07% sensitivity, and 95.14% specificity. Gu et al. [16] introduced an explainable breast cancer recurrence prediction model considering ML approaches LR, KNN, SVM, DT, RF, GBDT, MLP, XGBoost, etc., along with ensemble learning on the National Natural Science Foundation of China (NSFC) dataset. They resulted in 91.62% accuracy, 90.28% recall, and 89.39% F1-score. Lou et al. [17] purposed to compare the accuracy of forecasting models to predict recurrence within ten years after breast cancer surgery and to identify significant predictors of recurrence considering ML approaches including ANN, KNN, SVM, Naïve Bayesian Classifier (NBC), Cox Proportional-Hazards Regression (COX), etc. on The Cancer Genome Atlas (TCGA) datasets and resulted in 98.82% accuracy, 100% sensitivity, 100% specificity, 100% PPV, 99.08% NPV, 99.81% AUROC.

Magboo et al. [18] developed a breast cancer recurrence model considering ML approaches, including LR, NB, KNN, SVM, and PCA on WPBC datasets. They resulted in 80% accuracy, 80% precision, 62% recall, 76% F1-score, 0.81 AUROC, 0.62 AUPRC, and 0.3 Cohen kappa statistic. Alzu’bi et al. [19] developed a Natural Language Processing algorithm to extract key features about breast cancer from medical records at the King Abdullah University Hospital (KAUH) dataset considering the Term Frequency–Inverse Document Frequency (TF-IDF) method as the feature extraction technique and ML approaches including J48 DT, NB, Bagging, LR, SVM, KNN, MLP, PART, OneR, RF, etc. They resulted in 92.25% accuracy, 92.3% sensitivity, and 88.7% specificity. Zeid et al. [20] introduced an efficient, optimized framework for analyzing the performance of breast cancer model considering ML approaches LR, XGBoost, MLP, NB, RF, KNN, and DT on WBCD, WDBC, and WPBC datasets and resulted in accuracies of 98.3%, 99.2%, and 78.6%, AUCs of 99.3%, 99.5%, and 78.9%, precisions of 96.6%, 97.4%, and 77.7%, recalls of 97%, 97.4%, and 77.2%, and F1- scores of 96.7%, 97.4%, and 78% respectively. Ebrahim et al. [21] developed a model to predict breast cancer considering ML approaches including DT, LDA, LR, SVM, and ensemble techniques (ET) along with Probabilistic neural network (PNN), deep neural network (DNN), and recurrent neural network (RNN) on dataset taken from National Cancer Institute (NIH), USA and resulted in 98.7% accuracy, 96.7% precision, 76.4% recall, and 85.2% F1-score. Table 1 depicts the summary of the considered state-of-the-art works.

**Table 1:** Summary of the considered state-of-the-art works.

Ref	Techniques Employed	Dataset(s) Employed	Findings	Weaknesses
[8]	KNN, SVM, DT, MLP	WBCD and WDBC	Accuracy: 98.12%, Precision: 99.2%, Recall: 97.85%	Authors have limited performance parameters. More parameters should be employed including the Info Gain test, Gain Ratio test, and Chi-square tests
[9]	MLP, SVM, DNN, RNN	Breast Cancer Relapse Dataset (BCRD)	Accuracy: 94.53%	The rough neural network results in the lowest accuracy in comparison to other methods.
[10]	ML-DSS, RO	SEER	Accuracy: 86.0%, F-measure: 69.8%, Sensitivity: 67.1%, Specificity: 88.4%, AUC: 0.822	There is a need to improve the precision of the model through the weighting relative importance of the attributes by making a hybrid approach of ML algorithms and the models of RO.
[11]	MLP, SVM, SMO	WBCD	Accuracy: 96.99%, Precision: 97%, Recall: 97%, AUC: 0.968	More ML techniques should be considered to achieve enhanced predictive outcomes.
[12]	DT, SVM, MLP, KNN, LR, RF	Coimbra Breast Cancer Dataset (CBCD)	Accuracy: 100%, Precision: 100%, Recall: 100%, F1-score: 100%	The authors should have employed more models and parameters
[13]	MLP, CNN, SVM	EHRs	Precision: 81.47%, Recall: 77.82%, F1-score: 79.42%, AUC: 0.9489	The heterogeneity problem in clinical narratives should be addressed in the study.
[14]	SVM, ANN, NB, LDA	WDBC	Accuracy: 98.82%, Sensitivity: 98.41%, Specificity: 99.07%, AUC: 0.9994	SVM-LDA and NN-LDA outperform the other ML classifier models, but, NN-LDA is not chosen because of its longer time for computational.
[15]	LR, SVM, KNN, and PCA	UCI Sourced	Accuracy: 92.78%, Precision: 96.55%, Sensitivity: 91.07%, Specificity: 95.14%	This research work considers fewer variables in prediction.
[16]	LR, KNN, SVM, DT, RF, GBDT, MLP, XGBoost and Ensemble Learning	NFSC	Accuracy: 91.62%, Recall: 90.28%, F1-score: 89.39%	The authors have only designed the framework of a system and adopted existing methods.
[17]	ANN, KNN, SVM, NBC, COX	TCGA	Accuracy: 98.82%, Sensitivity: 100%, Specificity: 100%, PPV: 100%, NPV: 99.08%, AUROC: 99.81%	This research can be explored by designing the model of two-level or multi-level which will provide the effects of contextual volume of surgeon and hospital on the recurrence of breast cancer.
[18]	LR, NB, KNN, SVM and PCA	WPBC	Accuracy: 80%, Precision: 80%, Recall: 62%, F1-score: 76%, AUROC: 0.81, AUPRC: 0.62	SVM performance on imbalanced datasets is not very effective whereas on balanced datasets it is effective.
[19]	J48 DT, NB, LR, SVM, KNN, MLP, PART, OneR, RF and TF-IDF	KAUH sourced dataset	Accuracy: 92.25%, Sensitivity: 92.3%, Specificity: 88.7%	The unstructured and clinical variable format of data stored in the HER hospital increases the variability and complexity of their extraction.
[20]	LR, XGBoost, MLP, NB, RF, KNN, DT	WBCD	Accuracy: 98.3%, AUC: 99.3%, Precision: 96.6%, Recall: 97%, F1- score: 96.7%	This proposed work showed limited performance due to the imbalanced and small size of the dataset which leads to low prediction as compared with the classification of cancer on the other two datasets.
		WDBC	Accuracy: 99.2%, AUC: 99.5%, Precision: 97.4%, Recall: 97.4%, F1- score: 97.4%	
		WPBC	Accuracy: 78.6%, AUC: 78.9%, Precision: 77.7%, Recall: 77.2%, F1- score: 78%	
[21]	DT, LDA, LR, SVM, ET, PNN DNN, and RNN	NIH sourced dataset	Accuracy: 98.7%, Precision: 96.7%, Recall: 76.4%, F1-score: 85.2%	This proposed work would be more confirm the accuracy of the techniques of classification in the prediction of breast cancer considering the feature selection technique.

The critical findings from these considered similar state-of-the-art works can be stated as that the maximum of research includes only basic ML approaches considering a smaller number of performance parameters. Besides, working on imbalanced datasets without proper data pre-processing may not provide us a good predictive outcomes. Next, ensembling only conventional ML approaches needs a feature selection technique, and integrating feature selection and feature extraction techniques may not be sufficient in achieving improved outcomes. As a consequence, we planned for hybridization of feature selection and optimization on conventional ML approaches to obtain enhanced predictive outcomes.

## Materials and methods

The considered datasets are discussed in this section. In addition, the feature selection and optimization techniques employed in this study are briefly discussed. This study’s various ML classification approaches are placed in the last subsection.

### Datasets employed and pre-processing

Many criteria were set before we began considering data for the experiments in the study. Many researchers in the field of breast cancer use data from the Wisconsin Diagnostic Breast Cancer dataset (WDBC) and the Wisconsin Prognostic Breast Cancer dataset (WPBC), both of which may be found in the UCI Machine Learning Repository [22, 23]. Both datasets were obtained from the University of Wisconsin Hospitals. These datasets are incredibly granular, consisting of features extracted from digitized photos. Every detail lines up with the visible cell nuclei in the picture. The following Table 2 is a summary of the data sets available. Each dataset has numerical characteristics or properties associated with each sample or classification pattern.

**Table 2:** Descriptions of dataset(s) employed.

Dataset	No. of Instances	No. of Attributes	Missing Values	Attribute Type	Classes	Distribution of Class
WDBC	569	32	No	Real	Benign (B), Malignant (M)	357 (B), 212 (M)
WPBC	198	34	4	Real	Non-Recurrence (N), Recurrence (R)	151 (N), 47 (R)

The WDBC dataset is an extremely lean data clump made up of information mined from digitized photographs. This collection contains 569 example records, each containing 32 attributes (ID, Diagnosis, and 30 real-valued variables). All 30 input features allow for linear separation of the data set. All the details align with what can be seen in the photograph, which are the characteristics of cell nuclei. The first characteristic is a patient’s identification, and the second is a label for whether the patient’s cancer is malignant or benign. Calculated attributes for each cell nucleus fall in the 3–32 attribute range [24]. The radius equals the mean distance from the entrance to every other point around the perimeter. The variance in grayscale values is what we call the texture parameter. The smoothness characterizes the regional variation in radius length. The formula for the compactness factor is as follows: (area squared -1.0)2/perimeter2. The fractal dimension equals (a rough estimate of the coast)-1, and concavity describes the degree to which a contour is concave. Average, standard deviation and worst case are calculated across 30 characteristics. Field 3 reflects, for instance, the average radius, field 13 the standard deviation, and field 23 the worst radius. Features of the WDBC dataset indicate that there are three columns and three values (mean, standard error, and worst) for these characteristics.

The type and progression of breast cancer both impact the outlook for survival. A total of 198 observations and 47 recurrences (151 of which are not) make up the WPBC dataset. Like the other collections, the WPBC dataset includes both healthy and cancerous samples. The following are features of this data set: The first component is the patient’s unique ID. As for the second characteristic, it’s the output class: R for "recurrence" and N for "Non-Recurrence." Time is the third feature, and it describes the interval between episodes for "R" and "being healthy" for "N." Radius, area, perimeter (dimensions and shape of a nucleus), concavity, concave points, symmetry, fractal dimension (approximation of a coastline), compactness, texture, standard deviation of grayscale values, and smoothness (local variation in radius lengths) are the ten computed real values that the attributes 3–33 identify for cell nuclei. Tumor size, measured in centimeters, is the 34th feature. There are four distinct sizes of tumors. T-1 is less than two centimeters in length. The dimensions for a T-2 are between 2 and 5 centimeters. T-3 is longer than five centimeters. Any tumor that has ulcerated the skin or is connected to the chest wall is classified as T-4. Lymph node status is the number of malignant lymph nodes found during surgery. Lymph node status, or the number of auxiliary lymph nodes where cancer was found during surgery, is the 35th characteristic. Axillary lymph nodes, located in the armpit, are a primary site of metastasis for breast cancer. Lymph node status was missing as a value in four different records. Absent in four different files [25].

For ML models to make sense of visual input, data pre-processing is essential for any classification system. Using Data Preprocessing, we clean up the data set so that only accurate information is delivered. For optimal categorization results, ensure that your data is complete, accurate, and free of ambiguity. Errors and gaps in the data set can be remedied through preprocessing. In order to obtain clean data that is model-ready, the pre-processing stage is utilized to improve the quality of the dataset. The dataset included redundant and irrelevant information because it was compiled from many sources. We employ data cleansing methods to ensure that our data is free of such inconsistencies. Several pre-processing methods were applied to the Breast cancer dataset before classification tasks were performed using ML methods. The dataset was made more presentable during preprocessing by eliminating duplicates, missing values, and unnecessary layers. These procedures, which include a thorough cleaning, are necessary for getting the dataset ready for usage with machine learning models. It improves performance by removing unnecessary data characteristics. Several stages make up the pre-processing technique, each of which is described in turn below.

Noise in data is reduced, and missing values are handled during data cleaning. Get rid of blanks: The dataset was analyzed and used in this study [20]. Since the WPBC dataset has some missing and irrelevant data while the WDBC dataset does not, we clean the data by substituting the proper values for the missing ones. One attribute value (represented by "?") is missing in four different WPBC instances. The attribute entails supplying missing data for all instances of a given class. Getting rid of outliers indicates they were particularly destructive. They significantly affect a model’s predictions when using machine learning. To identify whether an outlier record is the consequence of a data collecting error or a unique event taken into account during data processing, researchers typically examine the records in question. An outlier is a statistic that doesn’t fit in with the rest of the numbers. It’s possible that removing outliers will result in a smaller dataset overall, but one that is nonetheless accurate. The analysis of statistical correlation eliminates the need to go into superfluous details. A common irrelevant aspect between the WPBC and the WDBC is the ’Sample code number,’ which is disregarded because it does not influence the categorization process. By starting the training process with scale-normalized features, data normalization shortens the total duration of the operation. Normalization aims to make the values of the features more comparable to one another.

### Feature extraction technique: Recursive Feature Elimination (RFE)

It is a method for selecting features based on their statistical significance that involves iteratively selecting features. The degree of statistical significance (p) is determined using the criteria for hypothesis testing. In hypothesis testing, the p-value is a statistical measure that represents the observed significant value of the input characteristic [26]. If the p-value of a certain input characteristic is less than the significance threshold (ρ), then there is a statistical link between the input and output features. The value of ρ is 0.05 for RFE. Starting with the dataset (D) having input feature set as {*f*_1_,*f*_2_,*f*_3_,….,*f*_*n*_}. The algorithm recursively deletes the features based on two selected hypotheses *Null hypothesis* $(H_{0})\;$ and *Alternative hypothesis $\left(H_{a}\right)$* as per Eqs (1) and (2) respectively.

Null Hypothesis ${(H}_{0})$: This hypothesis states that the feature set from the dataset (D), a subset of the feature set having will statistical importance (p) having conditions as per Eq 1 will be removed from the feature set.


$$H_{0}\to \;p\geq \;\rho \;(0.05)$$ (1)

Alternative Hypothesis ${(H}_{a})$: The feature set having statistical importance as per Eq 2 will be removed from the dataset.


$$H_{a}\to p<\rho \;(0.05)$$ (2)

The p-value can be calculated using the logistic regression model for each feature in the feature set of dataset D. The p-value can be calculated using Eq (3) or Eq (4).

$$p_{f_{i}}=\text{log}\left(\frac{p}{1-p}\right)$$ (3)

$$p_{f_{i}}=\sigma_{0}+\;\sigma_{i}m$$ (4)

Where fi is the selected feature for calculating the p-value, p is the probability of the selected feature, *σ*_0_, *σ*_*i*_ are the logistic regression parameters, and m is the value of selected feature fi.

### Optimization technique: Grey Wolf Optimization (GWO)

**Fig 1:**
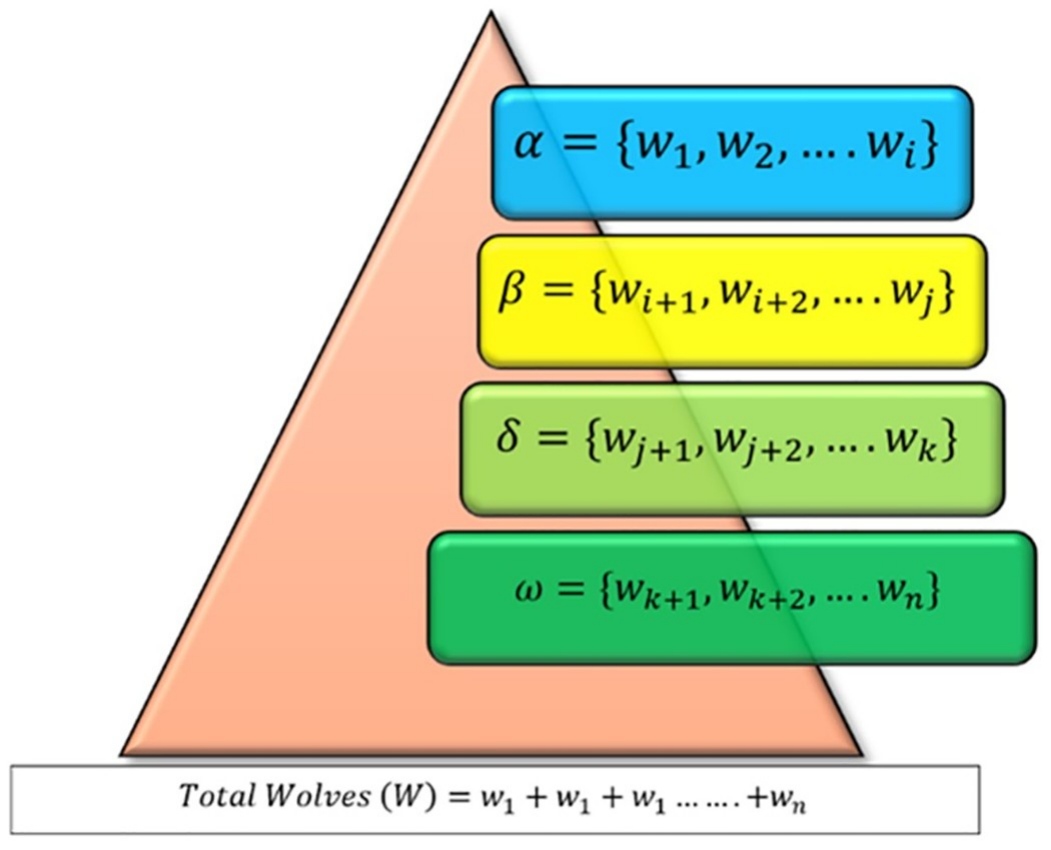
Social hierarchy of grey wolves.

Grey Wolf Optimization (GWO) is an algorithm that imitates the social structure and hunting skills of wild grey wolves to discover optimal solutions to optimization problems. GWO is a metaheuristic optimization method that combines swarm intelligence with swarm algorithms, much to PSO and GA [27]. The intricate social organization and astute hunting strategies of the grey wolf were the driving forces behind GWO. Most of the time, grey wolves are the most dominant predators in the areas where they live. The average number of grey wolves in a group is five to twelve. The entire wolve group is divided into four different sub-groups such as: α, β, δ, and ω as shown in Fig 1. The wolf in α group is the dominant wolf in the group and guides the others in activities like hunting, moving, and eating. In the absence of the leader wolf from the α group, whether due to illness or death, the strongest of the β wolves takes leadership. The wolves in δ and ω have less influence and power than α and β [28, 29]. The size of the above-said groups is formatted as Eq (5).


$$\left|\propto \right|<\left|\beta \right|<\left|\delta \right|<\;\left|\omega \right|$$ (5)

#### Mathematical model

**Fig 2:**
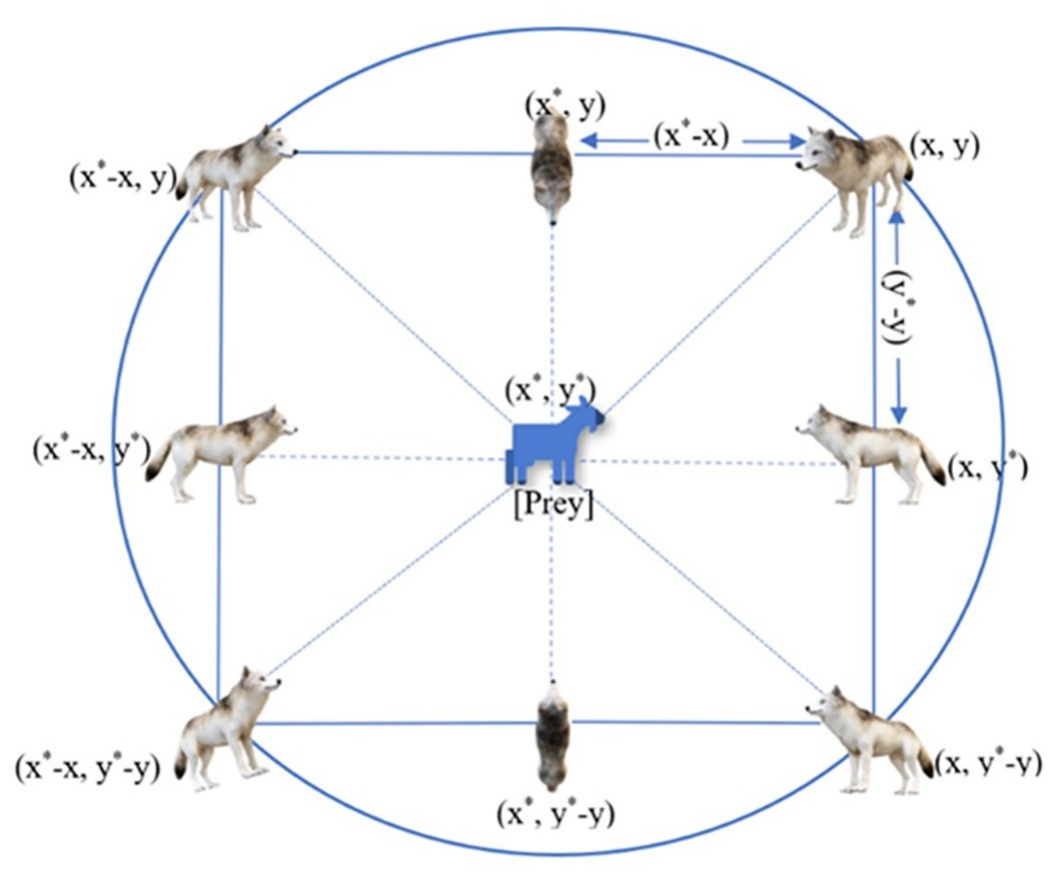
Position of wolves for encircling the prey.

Grey wolf social structure and hunting strategy (including tracking, encircling, and attacking) are modeled mathematically in this section using the GWO algorithm, as depicted in Fig 2.

#### Social structure

Eq (6) may be used to represent the results of the GWO algorithm’s attempt to mathematically model the grey wolf group’s hierarchical structure. The GWO algorithm uses α, β are used for hunting (for optimization), followed by δ and ω.


$$Solution=\left\{\begin{array}{ll} \alpha , & Best\;Solution\\ \beta ,\delta , & Average\;Solution\\ \omega , & Others \end{array}\right.$$ (6)

#### Encircling

For hunting the prey, the grey wolves initially encircle the prey. The encircling phase of the wolves can be represented by Eq (7).


$$\vec{D}=\left|\vec{C}*\vec{P_{t}}-\vec{W_{t}}\right|$$ (7)

Here, $\vec{P_{t}}$ and $\vec{W_{t}}$ is a vector representing the position of the prey respectively. $\vec{C}\;$ is the coefficient vector ranging [–1, 1] as represented in Eq (10). $\vec{D}$ is the calculated distance for updating the position of the wolf, and t shows the number of iterations. The position vector of the wolf and the prey can be represented as position vectors (x, y). The new position of the grey wolf by using Eq (8).


$${\vec{W}}_{t+1}=\vec{W_{t}}-\vec{A}*\;\vec{D}$$ (8)

$$\vec{A}=2*\vec{a}*{\vec{\tau }}_{1}-\vec{a}$$ (9)

$$\vec{C}=2*{\vec{\tau }}_{2}$$ (10)

Here, $\vec{A}$ is a coefficient vector ranging from [0,1] which can be calculated using Eq (9). ${\vec{\tau }}_{1}\;and\;{\vec{\tau }}_{2}$ are two random vectors in the range [0,1]. However, $\vec{a}$ is the vector set linearly decreasing from 2→0. In order to represent the hunting behavior of grey wolves, it is believed that α (the most likely solution), β, and δ know more about where the prey may be lurking. The algorithm keeps track of the best three solutions it has identified so far and forces the others (the ω wolves) to adjust their location accordingly. The distance vector for wolves from α, β, and ω can be represented as Eqs (11)–(13). Accordingly, the wolves from each group can be updated as Eqs (14)–(16), respectively.


$${\vec{D}}_{\propto }=\left|{\vec{C}}_{1}*\vec{W_{\propto }}-\vec{W}\right|$$ (11)

$${\vec{D}}_{\beta }=\left|\vec{C}*\vec{W_{\beta }}-\vec{W}\right|$$ (12)

$${\vec{D}}_{\omega }=\left|\vec{C}*\vec{W_{\omega }}-\vec{W}\right|$$ (13)

$${\vec{W}}_{\propto +1}=\vec{W_{\propto }}-{\vec{A}}_{1}*\;{\vec{D}}_{\propto }$$ (14)

$${\vec{W}}_{\beta +1}=\vec{W_{\beta }}-{\vec{A}}_{2}*\;{\vec{D}}_{\beta }$$ (15)

$${\vec{W}}_{\delta +1}=\vec{W_{\delta }}-{\vec{A}}_{3}*\;{\vec{D}}_{\delta }$$ (16)

#### Attacking

A wolf may follow its prey anywhere within a hypersphere. But that’s still not enough to replicate the grey wolf’s social intelligence. As was previously said, social hierarchy is crucial to the success of a pack’s hunt and its ability to stay alive. The α, β, and ω solutions are thought to be the best for simulating social hierarchy. For the purpose of simplicity, GWO assumes that there is only one answer for each class, even if, in nature, there may be more than one wolf in each category. Given that α, β, and ω are the best answers in the population, it is plausible to believe that they know where the global optimum of optimization issues is. As a result, the other wolves need to revise their strategies as Eq (17).


$${\vec{W}}_{\delta +1}=\frac{\vec{W_{\propto }}+\vec{W_{\beta }}+\vec{W_{\delta }}}{3}$$ (17)

#### Exploration and exploitation

When optimizing a task, an algorithm may exhibit both exploratory and exploitative tendencies. To avoid being stuck in a local optimum, the algorithm’s exploration phase involves making unexpected modifications to the solutions in an effort to find previously unexplored regions of the problem’s search space. Exploitation aims to refine the predicted results from the exploration phase by learning about the area around each solution. Therefore, solutions should be tweaked incrementally to converge to the global optimum. The major problem is that exploitation and exploration often go against one another. As a result, for an algorithm to efficiently estimate the global optimum of a problem, it must be able to take into account and strike a compromise between these competing behaviors during optimization.

### Classification techniques employed

The selection of seven ML methods in this study for breast cancer and breast cancer relapse prediction reflects a deliberate strategy to harness the strengths of diverse algorithms and enhance the robustness of our predictive models. The likelihood of breast cancer recurrence can be predicted using one of several different categorization systems [30]. ML and statistical approaches classify patients into benign and malignant or relapse and non-relapse groups using their medical histories, genetic profiles, and clinical data. Several ML classification methods, including the RFE and GWO, are used in the dataset, including the NB, KNN, LR, SVM, MLP, RF, and DT [31, 32]. These seven ML methods were chosen to provide a comprehensive exploration of different modeling paradigms. NB works as a probabilistic classifier and performs conditional probability, KNN considers local data neighborhoods, LR offers simplicity and interpretability, SVM excels in handling complex relationships, MLP captures intricate patterns, RF provides robustness against overfitting, and DT offers interpretability. These diverse methods allow us to account for various aspects of the complex breast cancer landscape, ensuring a more holistic understanding and accurate prediction of breast cancer outcomes.

## Proposed model

**Fig 3:**
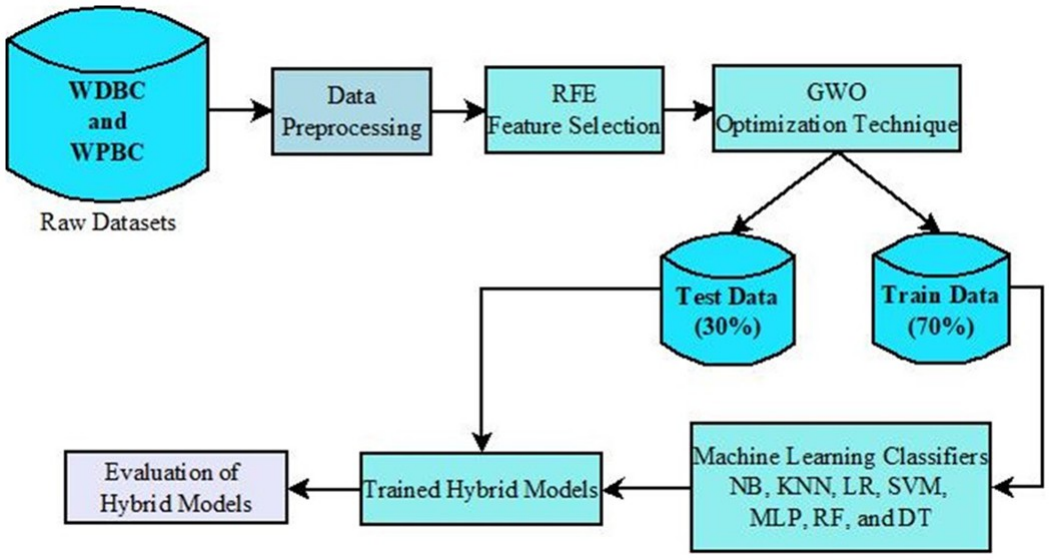
Proposed work block diagram.

The reported model uses two types of Breast cancer relapse datasets. Fig 3 shows the workflow of the proposed model. Initially, the datasets undergo a preprocessing step to handle the outliers present in them. The RFE feature selection algorithm is applied to the processed dataset to identify the correlated features. The GWO optimization algorithm is then applied to the featured dataset to bring the optimized number of features into the front without hampering the dataset’s utility. Finally, seven different ML classifiers are applied to evaluate the performance of the proposed model with six different evaluative measures. Algorithm 1 shows the pseudocode of the proposed model.

**Algorithm 1: Pseudocode of the Proposed Work**

**Input:** Dataset (D): {f_1_, f_2_, f_3,……,_ f_k_}, number of features k, maximum iteration N, Number of Wolves (n)

**Output:** Performance of the model using different evaluative parameters.

 • Preprocess the raw dataset to obtain D

 • Apply RFE to the preprocessed dataset D

  • Define $H_{0}$ and $H_{a}$

  • for i← 1 to k

   • find p-value of f_i_ as $\text{log}\left(\frac{p}{1-p}\right)$

   • Determine the suitable hypothesis for the selected feature f_i_

   • If (${p\;\in H}_{0}$)

    • Discard f_i_

   • Else

    • *D*′ ← *f*_*i*_

   • End if

   • i←i+1

  • End for

 • Apply GWO to featured dataset *D*′

  • Initialize the population of wolves (W)

  • Define α, β, ω, ρ such that |*∝*| < |*β*| < |*δ*| < |*ω*|

  • Initialize the parameters (a, A, C)

  • Calculate Fitness function (F)

  • For ∀ f_i_ ∈ *D*′

   • While (t<N)

    • For i← 1 to n

     • Calculate distance vector $\vec{D}$

     • Determine the position of the search agent W_i_

     • Calculate the fitness function

    • End for

    • Update a, A, C

    • Find the best solution based on fitness function

    • Update the next position of *W*_*α*_, *W*_*β*_, *and W*_*ω*_ as ${\vec{W}}_{\propto +1}=\vec{W_{\propto }}-{\vec{A}}_{1}*\;{\vec{D}}_{\propto },\;\vec{W_{\beta +1}}=\vec{W_{\beta }}-{\vec{A}}_{2}*\;{\vec{D}}_{\beta },\;{\vec{W}}_{\delta +1}=\vec{W_{\delta }}-{\vec{A}}_{3}*\;{\vec{D}}_{\delta }$

    • Update the next position of *W*_δ_ as $\frac{\vec{W_{\propto }}+\vec{W_{\beta }}+\vec{W_{\delta }}}{3}$

    • t← t+1

   • End While

  • Update the optimal features in *D*′ to obtain optimal dataset *D*’’

  • End For

 • Apply ML classifiers to *D’’* for calculating the evaluative parameters.

## Results and discussion

Several presumptions are included in evaluating this suggested ML-based hybrid approach. The feature selection technique RFE and the optimization technique GWO were applied to seven conventional ML techniques to build new novel ML-based hybrid approaches by enhancing the evaluative measures [33, 34]. A workstation outfitted with 8GB of RAM, a 500GB SSD, a 1TB HDD, a 3.6GHz Intel Core i5 CPU, and Ubuntu 20.04 has been used to successfully test the proposed system. An extensive empirical study of the gathered results should be a part of any planned undertaking. Through a methodical experimental procedure, these measures seek to build a real-to-expected class confusion matrix. True positives and negatives are represented by the letters T_A_ and T_B_ in the confusion matrix, whereas false positives and false negatives are represented by the letters F_A_ and F_B_ [35–37]. Performance metrics for classification in this study include Accuracy (A_C_), Misclassification Rate (M_R_), Precision (P_R_), Sensitivity (S_N_), Specificity (S_P_), F1-Score (F_S_), False Negative Rate (F_NR_), False Positive Rate (F_PR_), Mathew’s Correlation Coefficient (M_CC_), and Balanced Accuracy (B_A_). Detailed formulations for these metrics are provided in Eqs (18)–(27).


$$A_{C}=\frac{T_{A}+\;T_{B}}{T_{A}+\;T_{B}+\;F_{A}+\;F_{B}}$$ (18)

$$M_{R}=\frac{F_{A}+\;F_{B}}{T_{A}+\;T_{B}+\;F_{A}+\;F_{B}}$$ (19)

$$P_{R}=\frac{T_{A}}{T_{A}+\;F_{A}}$$ (20)

$$S_{N}=\frac{T_{A}}{T_{A}+\;F_{B}}$$ (21)

$$S_{P}=\frac{\;T_{B}}{\;T_{B}+\;F_{A}}$$ (22)

$$F_{S}=\frac{{2\;\times \;T}_{A}}{{2\;\times T}_{A}+\;F_{B}+\;F_{B}}$$ (23)

$$F_{NR}=\frac{F_{B}}{T_{A}+\;F_{B}}$$ (24)

$$F_{PR}=\frac{F_{A}}{\;T_{B}+\;F_{A}}$$ (25)

$$M_{CC}=\frac{\left(T_{A}+\;T_{B}\right)-\;\left(F_{A}+\;F_{B}\right)}{\sqrt{\left(T_{A}+\;F_{A}\right)\left(T_{A}+\;F_{B}\right)\left(T_{B}+\;F_{A}\right)\left(\;T_{B}+\;F_{B}\right)}}$$ (26)

$$B_{A}=\frac{S_{N}+\;S_{P}}{2}$$ (27)

### Results analysis on WDBC dataset

**Fig 4:**
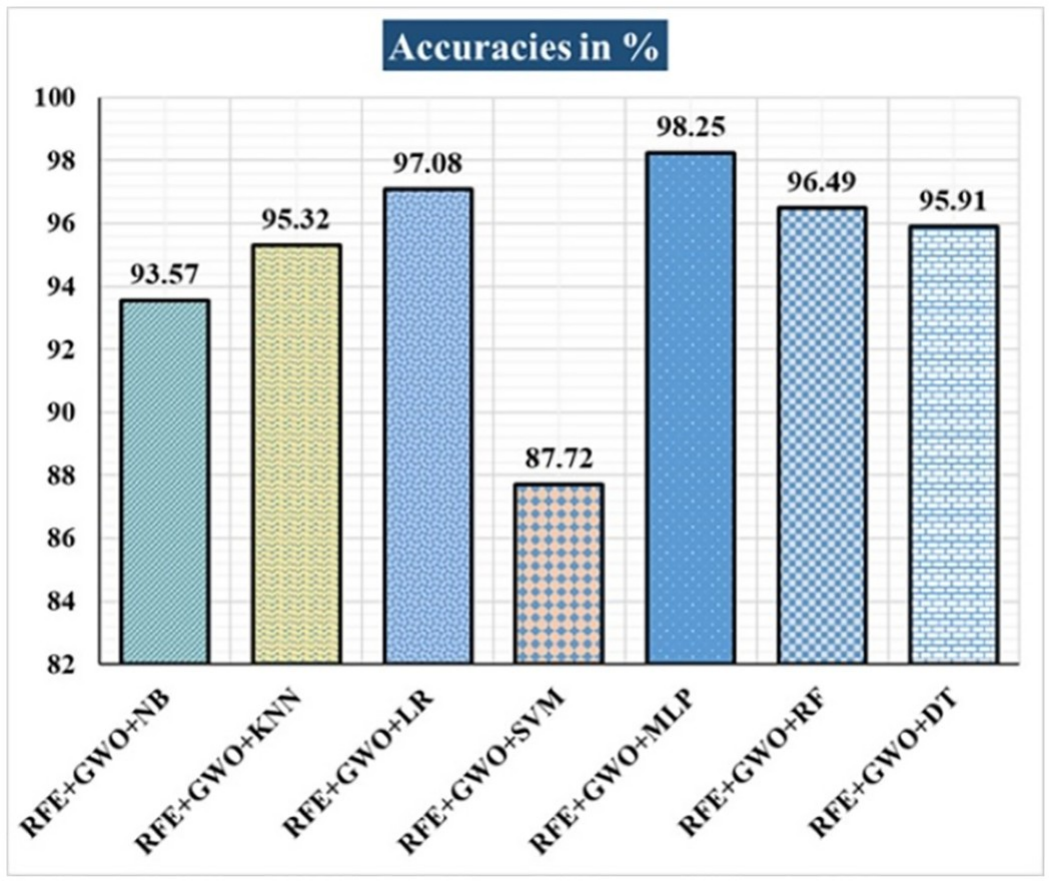
Recorded accuracies in % for the hybrid ML approaches on WDBC dataset.

The various ML-based hybrid approaches considered in this study employ the feature selection technique RFE, the optimization technique GWO, and seven conventional ML techniques, including NB, KNN, LR, SVM, MLP, RF, and DT. In the first, we applied these hybrid approaches to the WDBC dataset. Table 3 lists the results of extensive analyses of the performance of the proposed ML-based hybrid approaches. Figs 4 through 13 show the results obtained for A_C_, M_R_, P_R_, S_N_, S_P_, F_S_, F_NR_, F_PR_, M_CC_, and B_A,_ respectively, in percentage. According to the observations based on the performance measurements, the hybrid approach " RFE+GWO+MLP" outperforms all other six suggested hybrid approaches with an accuracy of 98.25%, precision of 98.13%, sensitivity of 99.06%, specificity of 96.92%, F1-score of 98.59%, etc. Besides, this hybrid ML approach also provides enhanced outcomes comparatively for other evaluative parameters considered in this work, including misclassification rate, FNR, FPR, MCC, and balanced accuracy, as shown in Table 3, which leads us to consider this as the recommended hybrid model for WDBC dataset. The ROC Curve and the AUC value for this recommended hybrid approach are calculated in the next, as depicted in Fig 14. The obtained AUC value of 0.982 for this recommended hybrid approach itself justifies the significance of this proposed approach on the WDBC dataset.

**Fig 5:**
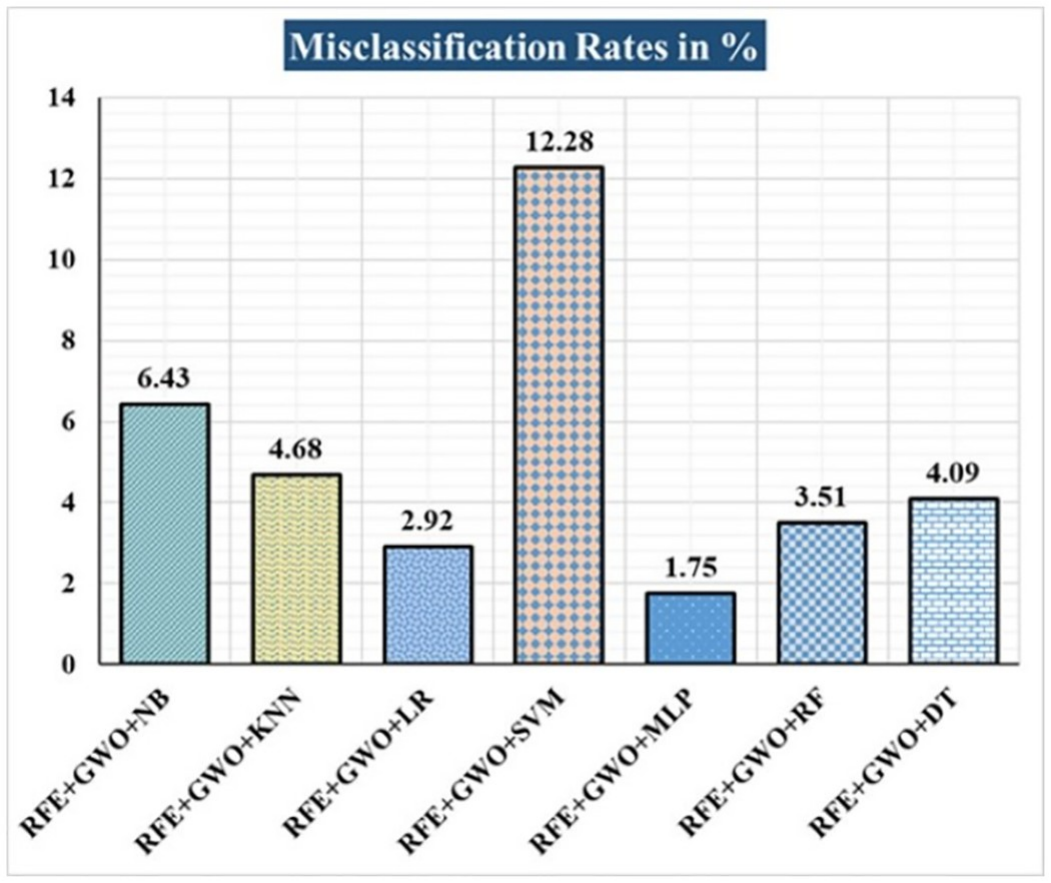
Recorded MCRs in % for the hybrid ML approaches on the WDBC dataset.

**Fig 6:**
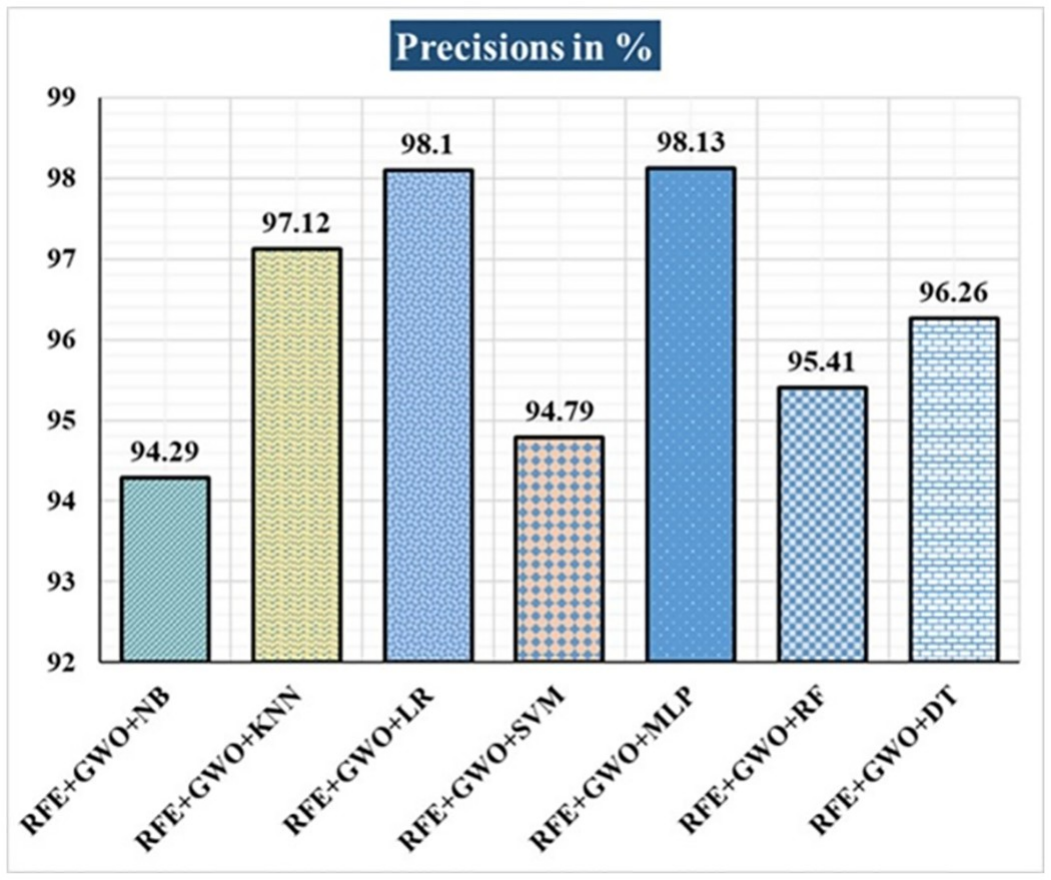
Recorded precisions in % for the hybrid ML approaches on the WDBC dataset.

**Fig 7:**
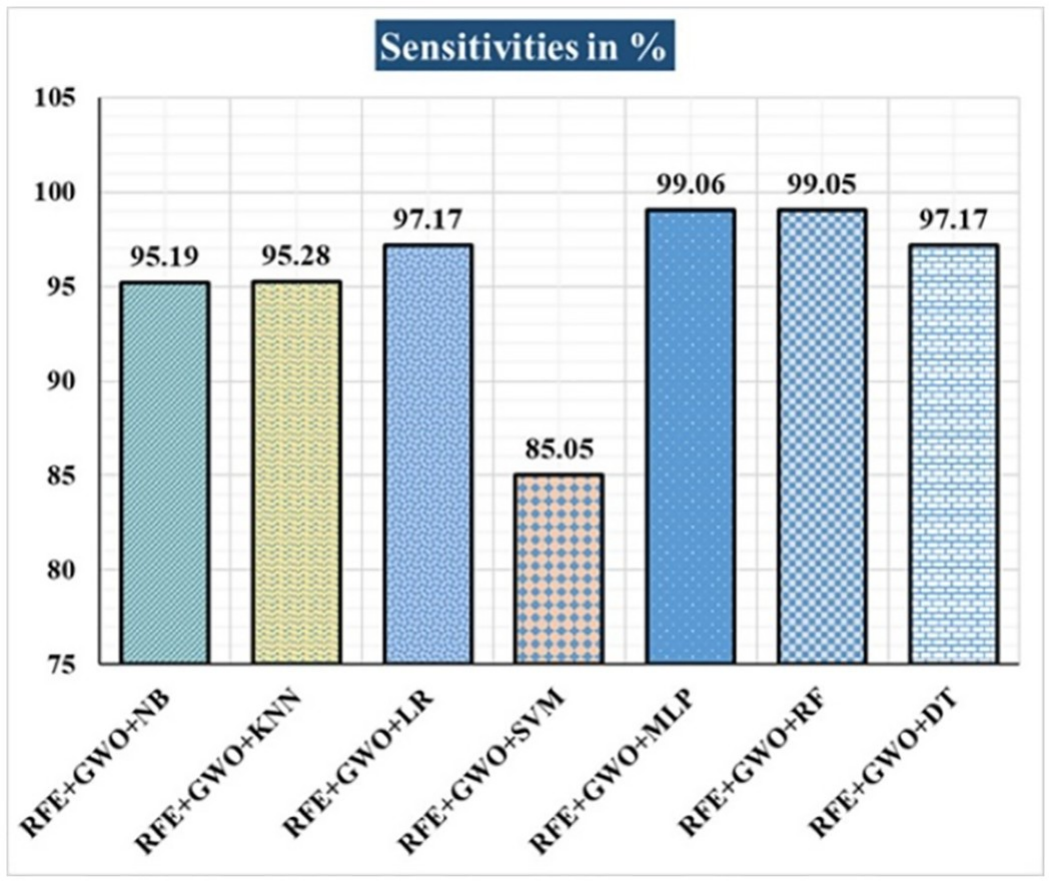
Recorded sensitivities in % for the hybrid ML approaches on the WDBC dataset.

**Fig 8:**
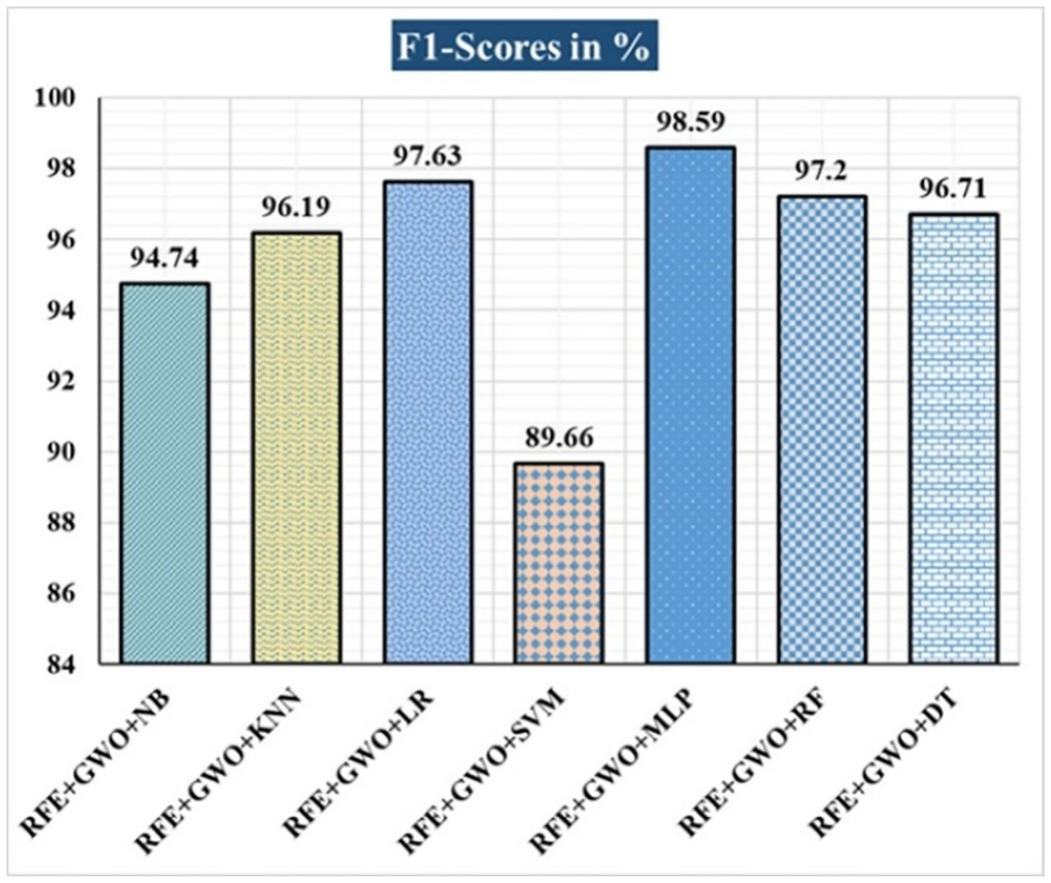
Recorded F1-scores in % for the hybrid ML approaches on WDBC dataset.

**Fig 9:**
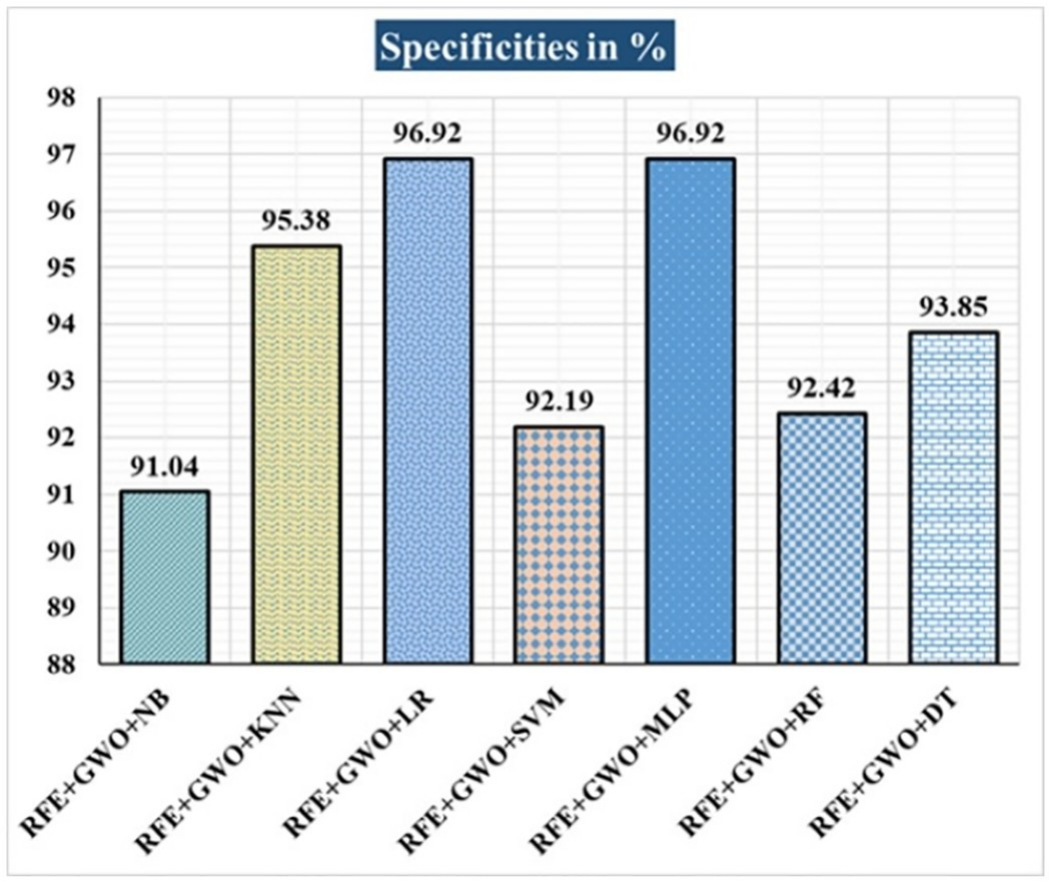
Recorded specificities in % for the hybrid ML approaches on the WDBC dataset.

**Fig 10:**
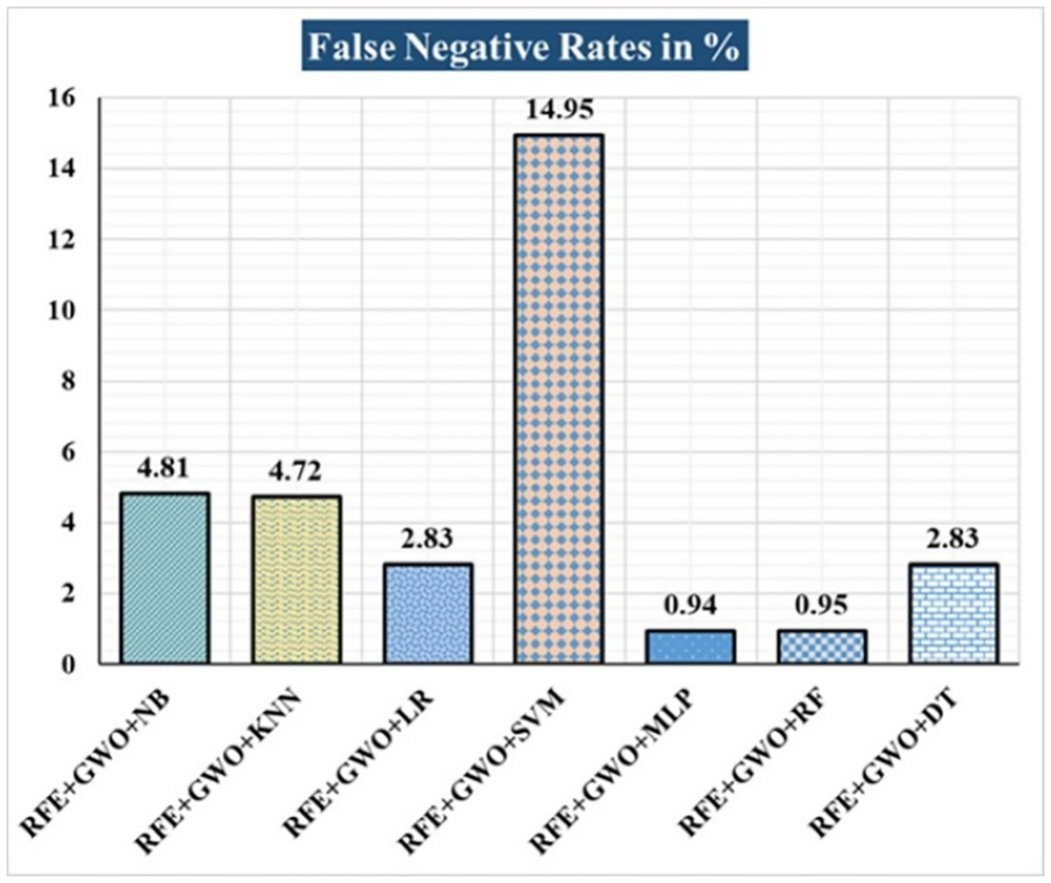
Recorded FNRs in % for the hybrid ML approaches on the WDBC dataset.

**Fig 11:**
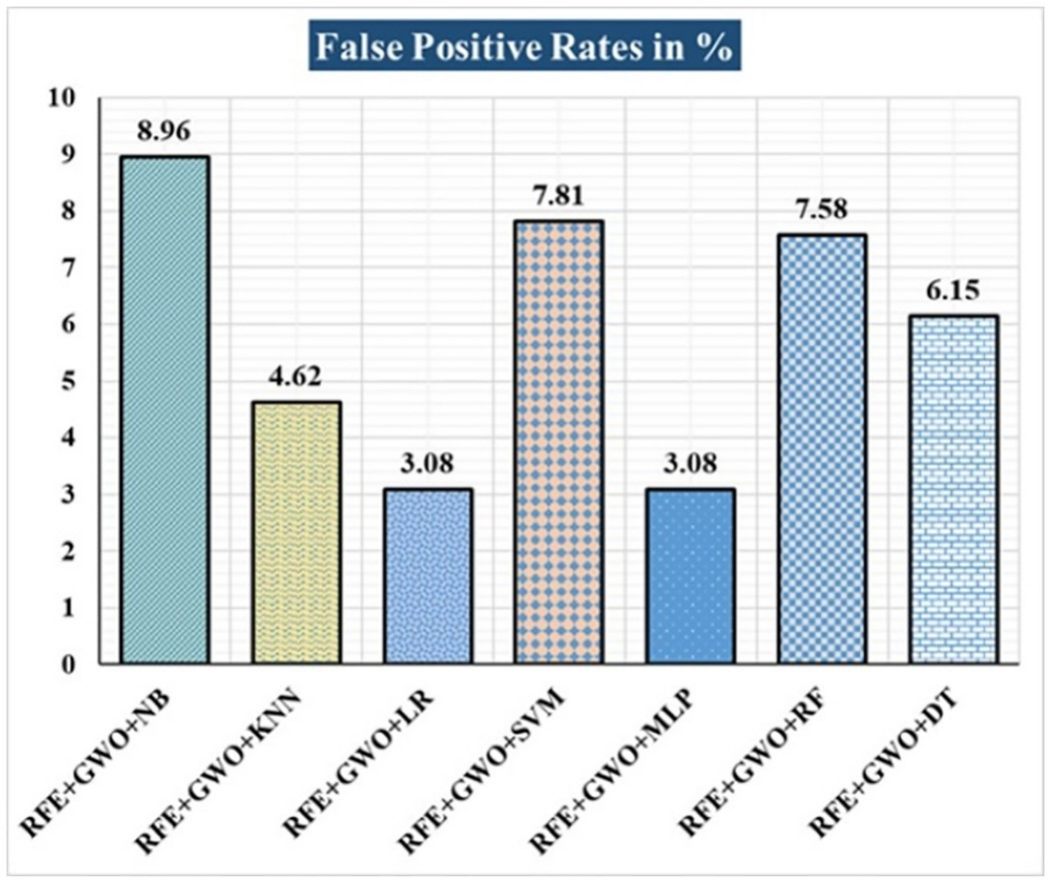
Recorded FPRs in % for the hybrid ML approaches on the WDBC dataset.

**Fig 12:**
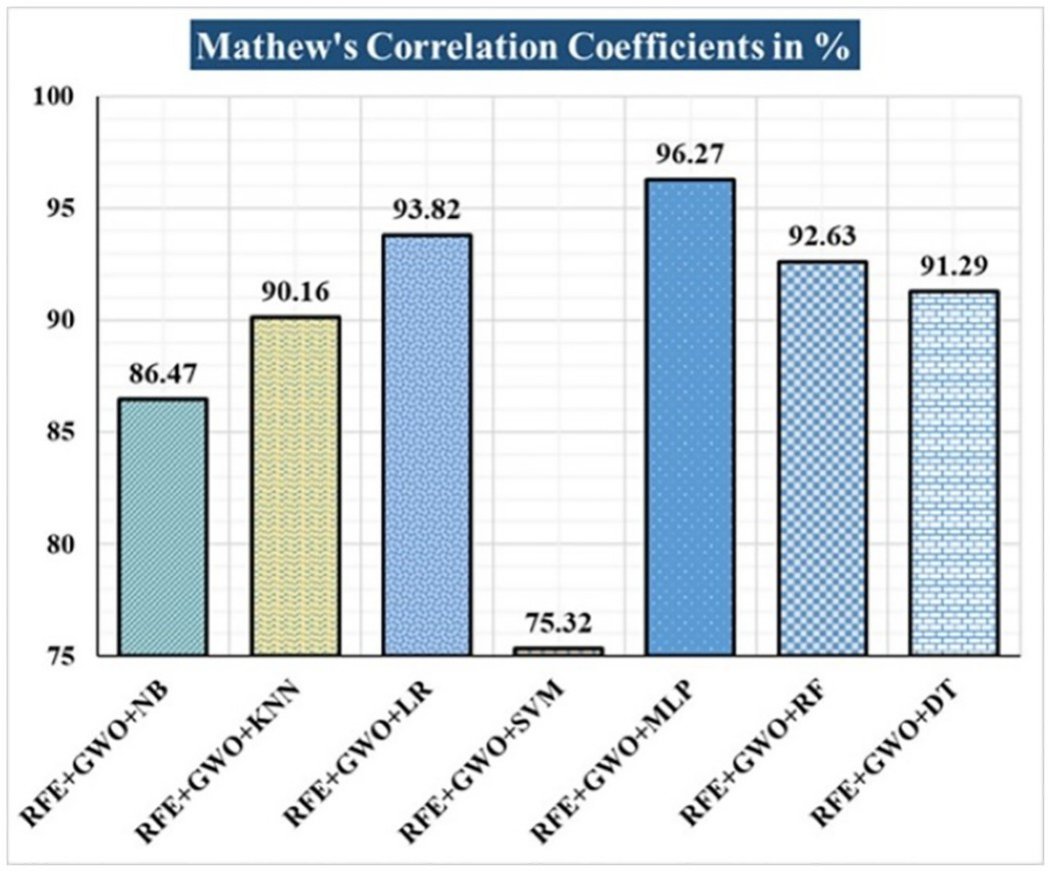
Recorded MCCs in % for the hybrid ML approaches on the WDBC dataset.

**Fig 13:**
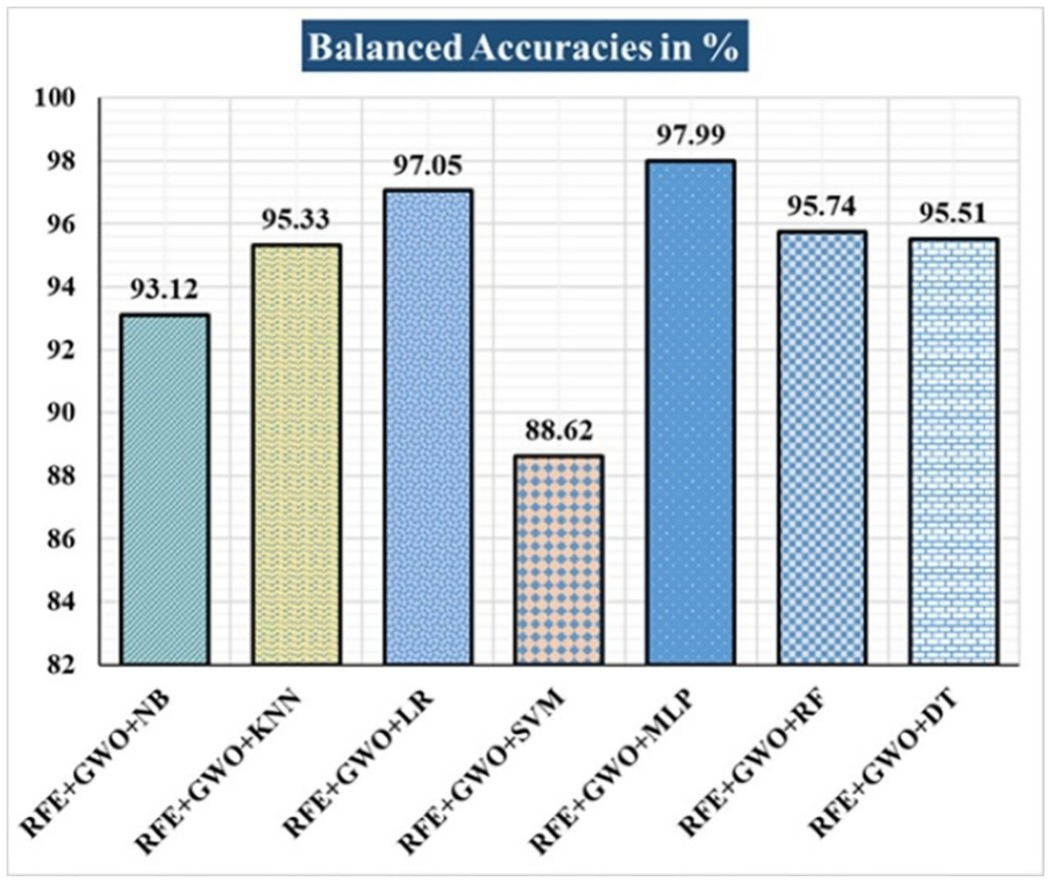
Recorded balanced accuracies in % for the hybrid ML approaches on the WDBC dataset.

**Fig 14:**
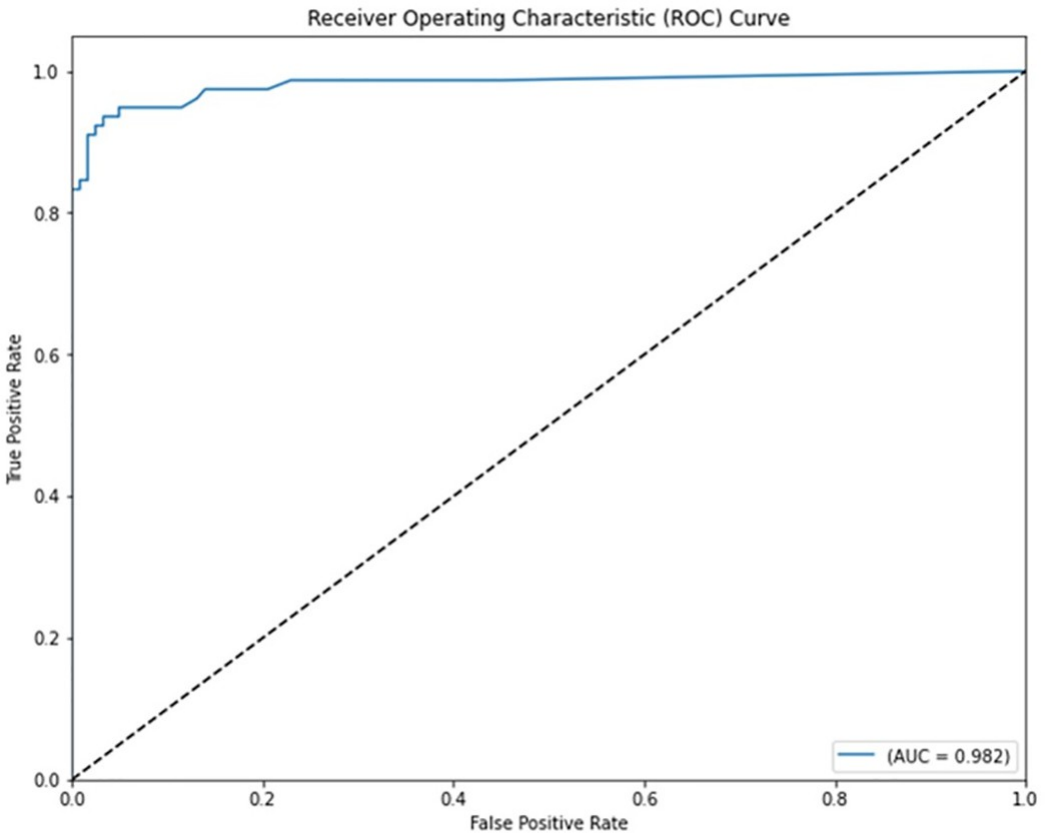
ROC curve with AUC value obtained employing the hybrid approach (RFE and GWO along with MLP) on WDBC dataset.

**Table 3:** Observed results employing various hybrid ML approaches on the WDBC dataset.

Hybrid ML Approaches	A_C_ in %	M_R_ in %	P_R_ in %	S_N_ in %	F_S_ in %	S_P_ in %	F_NR_ in %	F_PR_ in %	M_CC_ in %	B_A_ in %
RFE+GWO+NB	93.57	6.43	94.29	95.19	94.74	91.04	4.81	8.96	86.47	93.12
RFE+GWO+KNN	95.32	4.68	97.12	95.28	96.19	95.38	4.72	4.62	90.16	95.33
RFE+GWO+LR	97.08	2.92	98.1	97.17	97.63	96.92	2.83	3.08	93.82	97.05
RFE+GWO+SVM	87.72	12.28	94.79	85.05	89.66	92.19	14.95	7.81	75.32	88.62
RFE+GWO+MLP	98.25	1.75	98.13	99.06	98.59	96.92	0.94	3.08	96.27	97.99
RFE+GWO+RF	96.49	3.51	95.41	99.05	97.2	92.42	0.95	7.58	92.63	95.74
RFE+GWO+DT	95.91	4.09	96.26	97.17	96.71	93.85	2.83	6.15	91.29	95.51

### Results analysis on WPBC dataset

**Fig 15:**
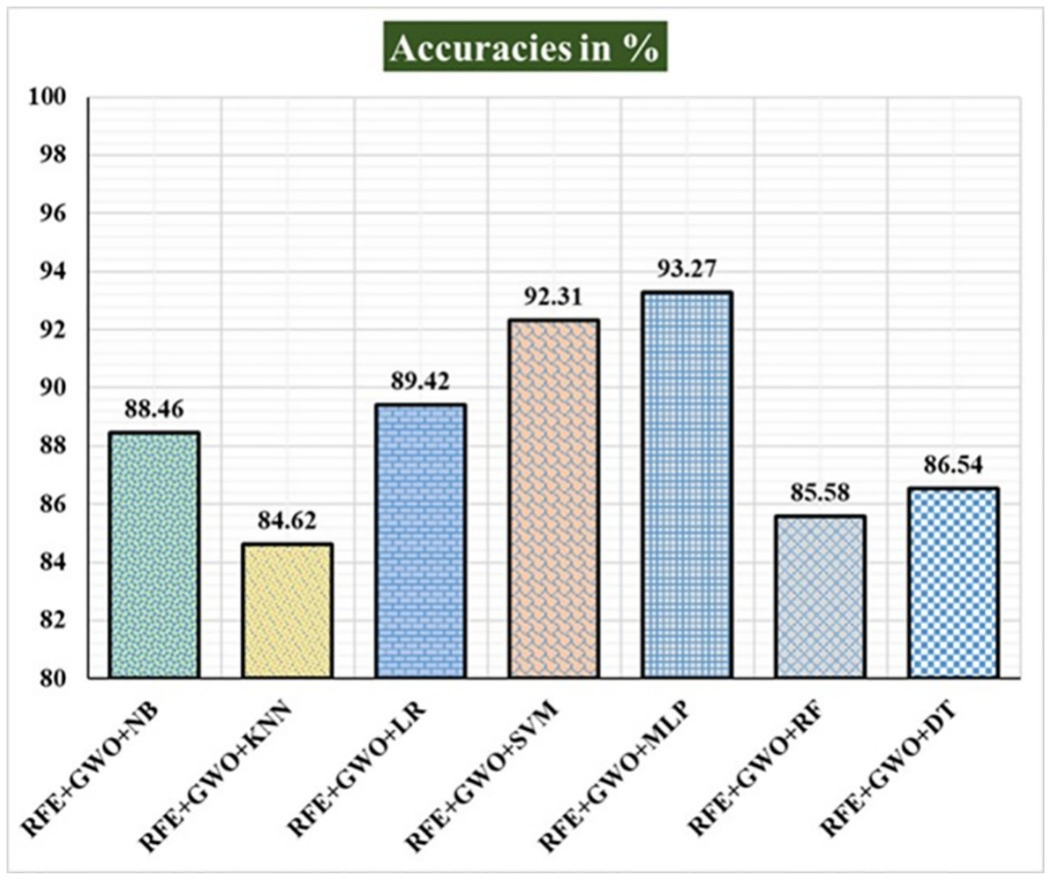
Recorded accuracies in % for the hybrid ML approaches on the WPBC dataset.

**Fig 16:**
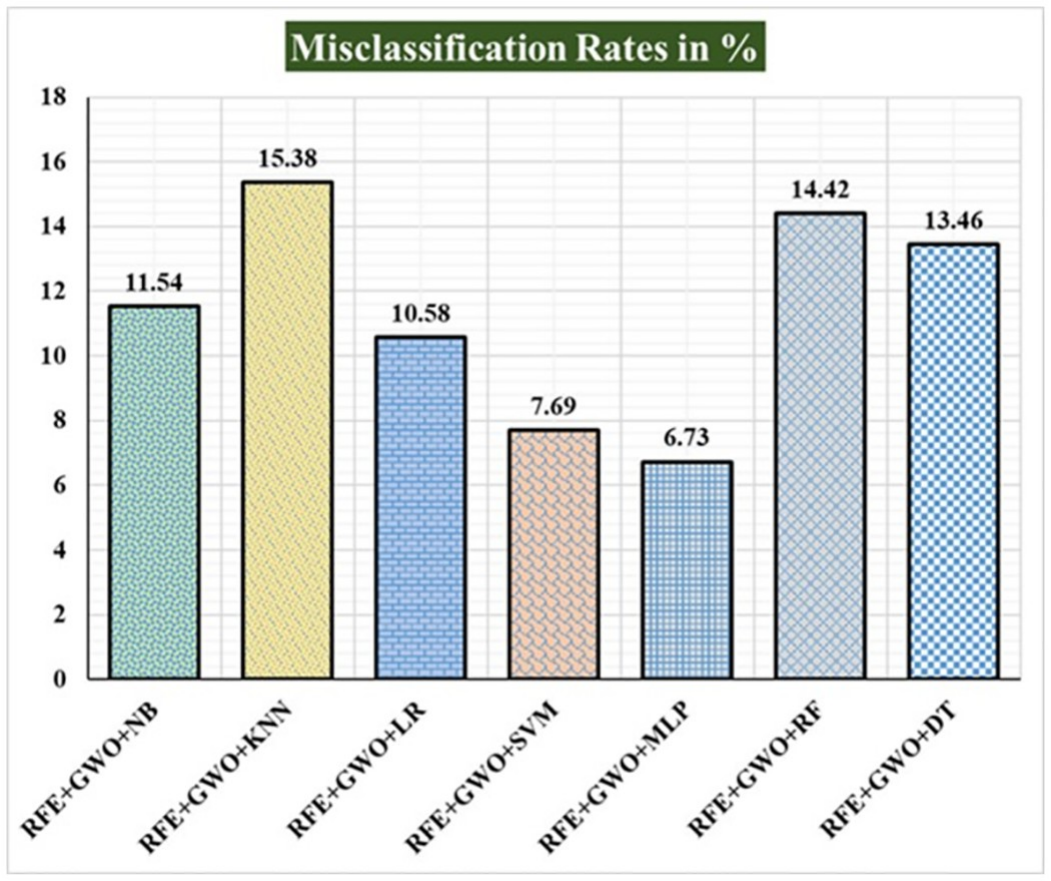
Recorded MCRs in % for the hybrid ML approaches on the WPBC dataset.

**Fig 17:**
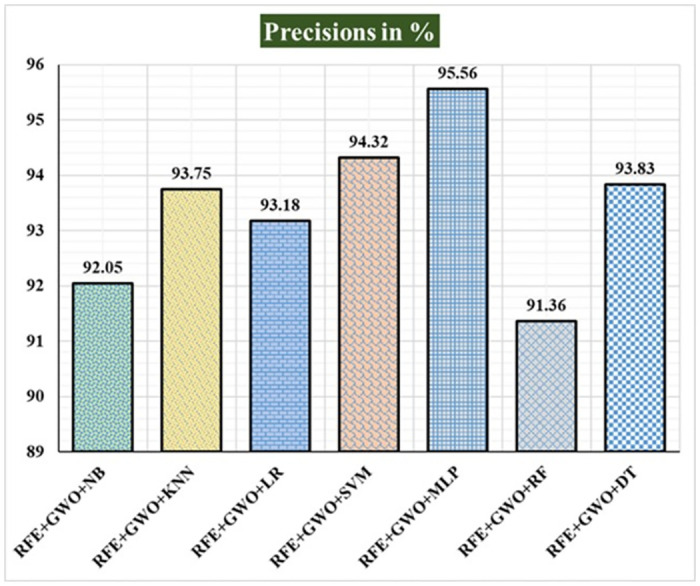
Recorded precisions in % for the hybrid ML approaches on the WPBC dataset.

**Fig 18:**
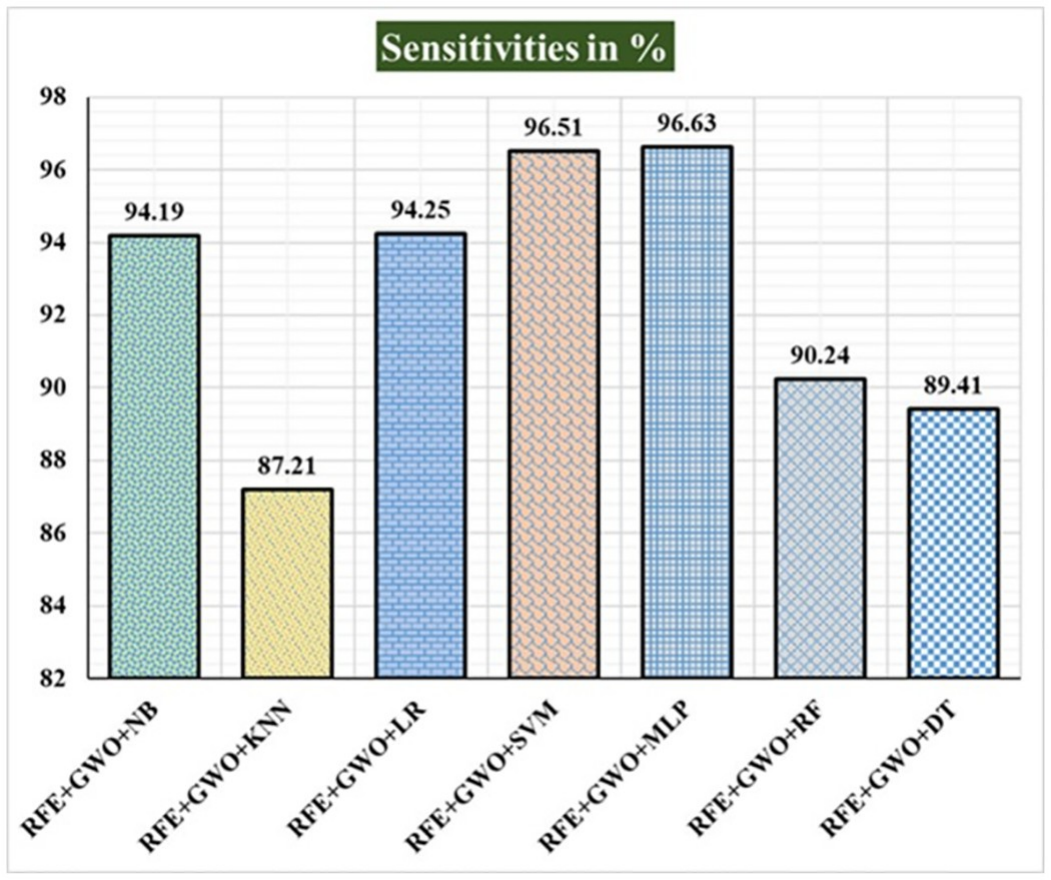
Recorded sensitivities in % for the hybrid ML approaches on the WPBC dataset.

**Fig 19:**
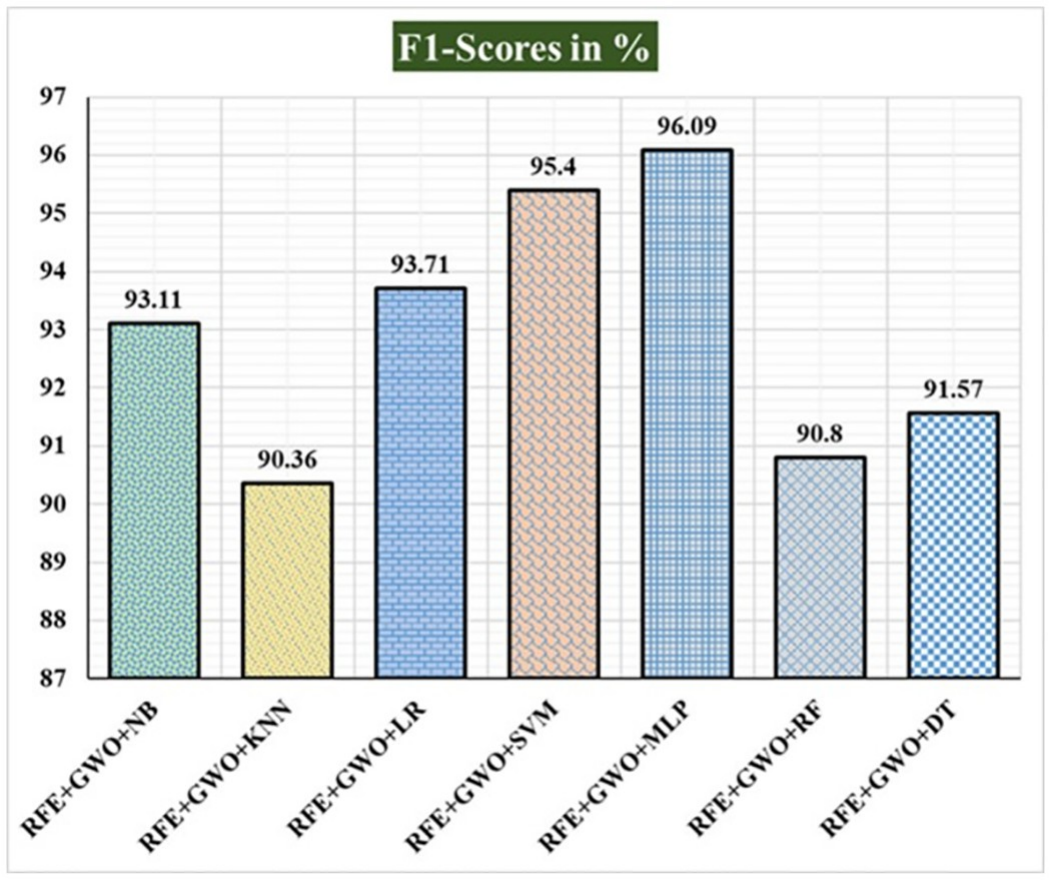
Recorded F1-scores in % for the hybrid ML approaches on the WPBC dataset.

**Fig 20:**
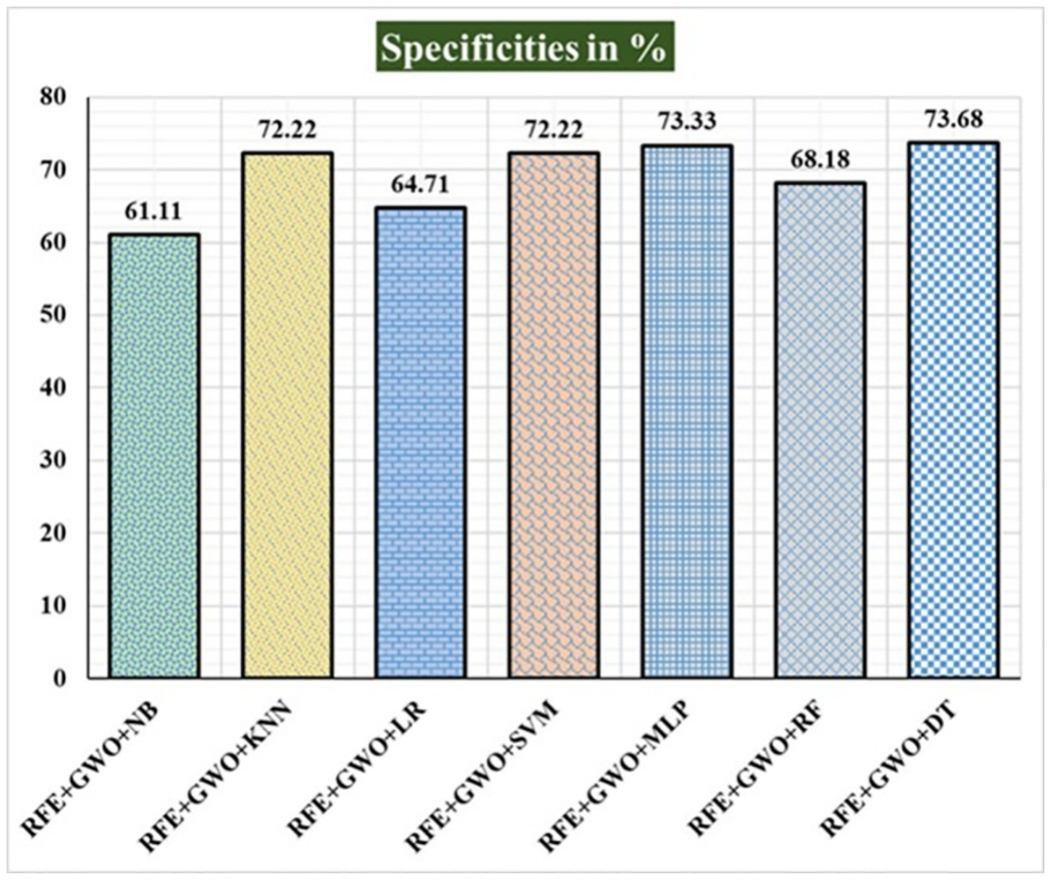
Recorded specificities in % for the hybrid ML approaches on the WPBC dataset.

**Fig 21:**
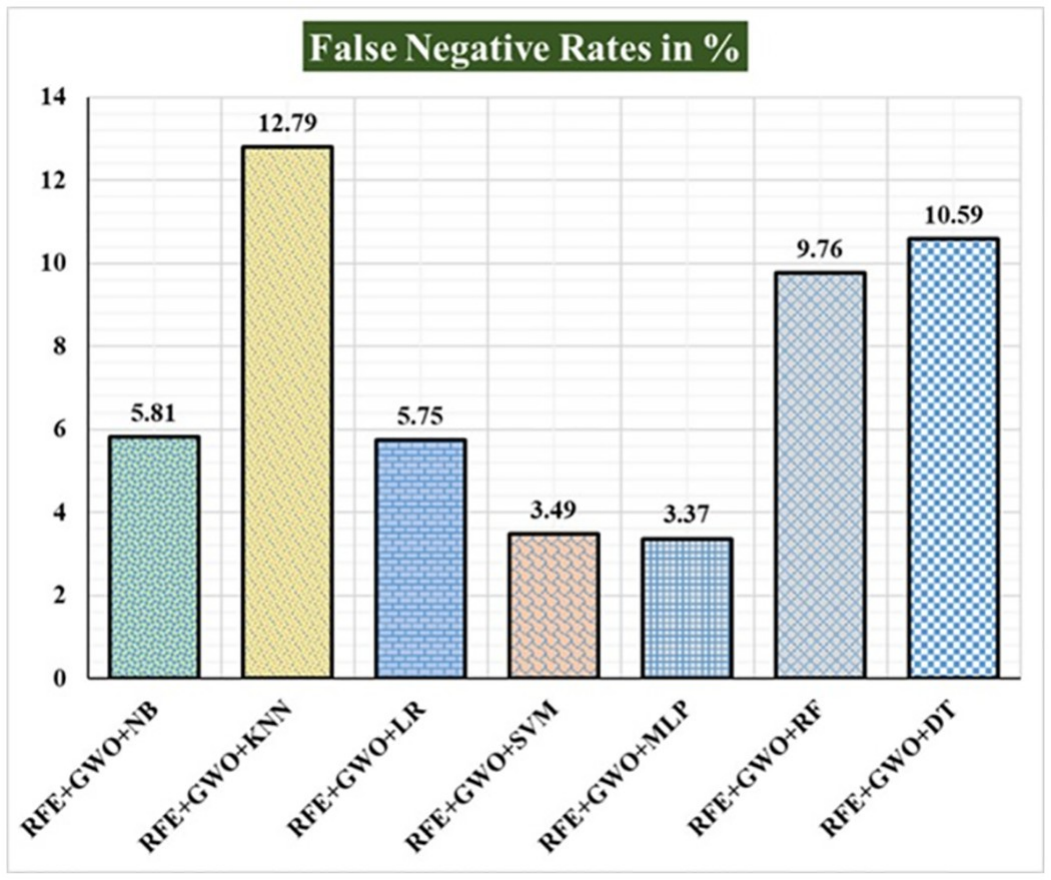
Recorded FNRs in % for the hybrid ML approaches on the WPBC dataset.

**Fig 22:**
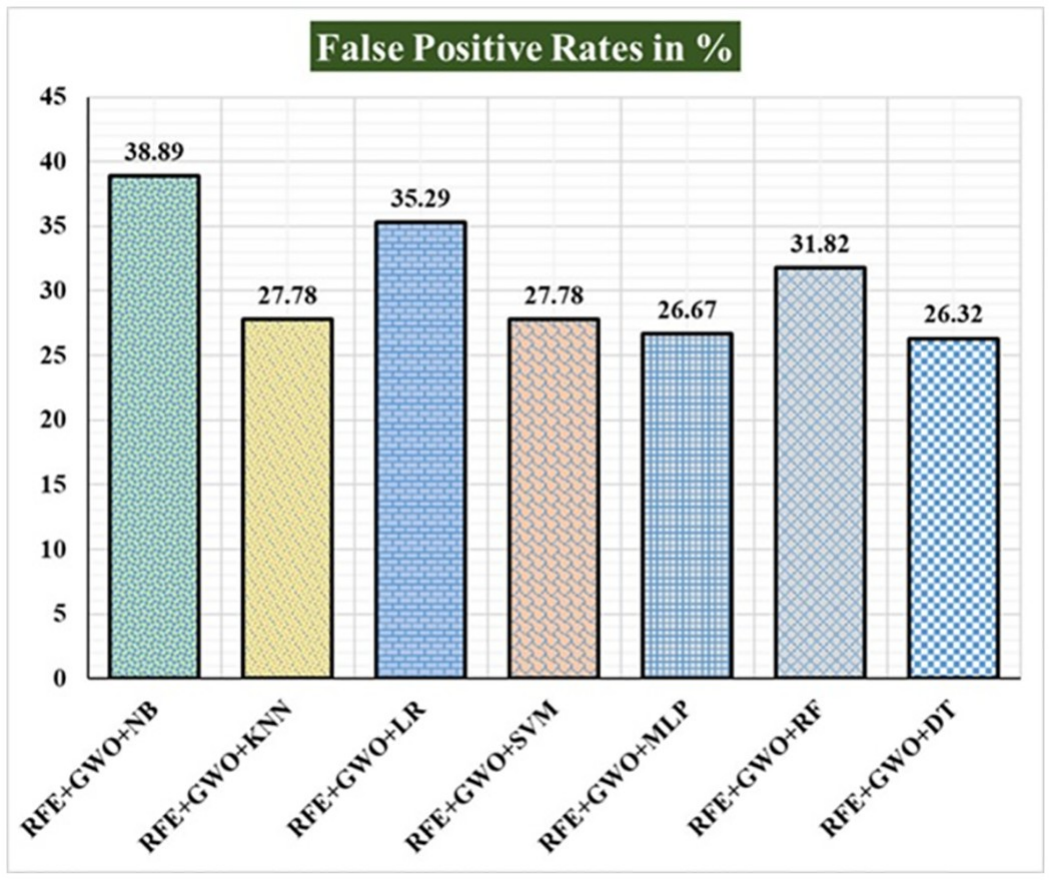
Recorded FPRs in % for the hybrid ML approaches on the WPBC dataset.

**Fig 23:**
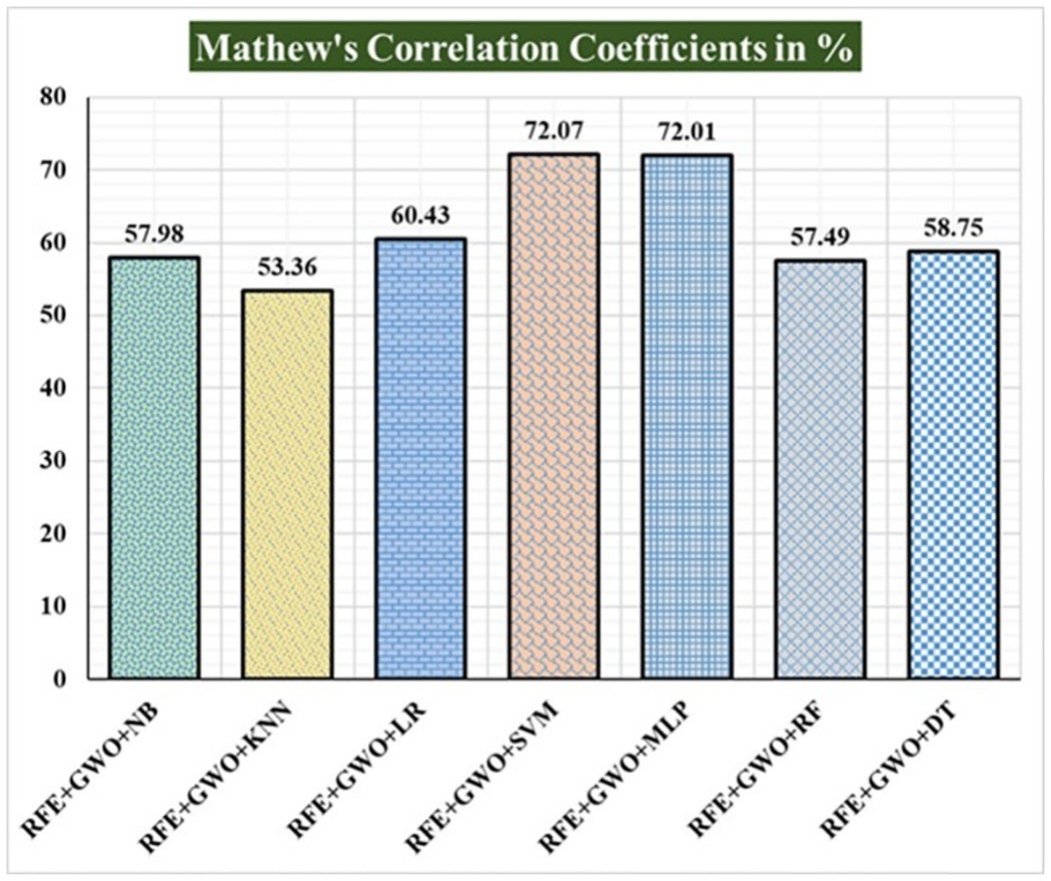
Recorded MCCs in % for the hybrid ML approaches on the WPBC dataset.

**Fig 24:**
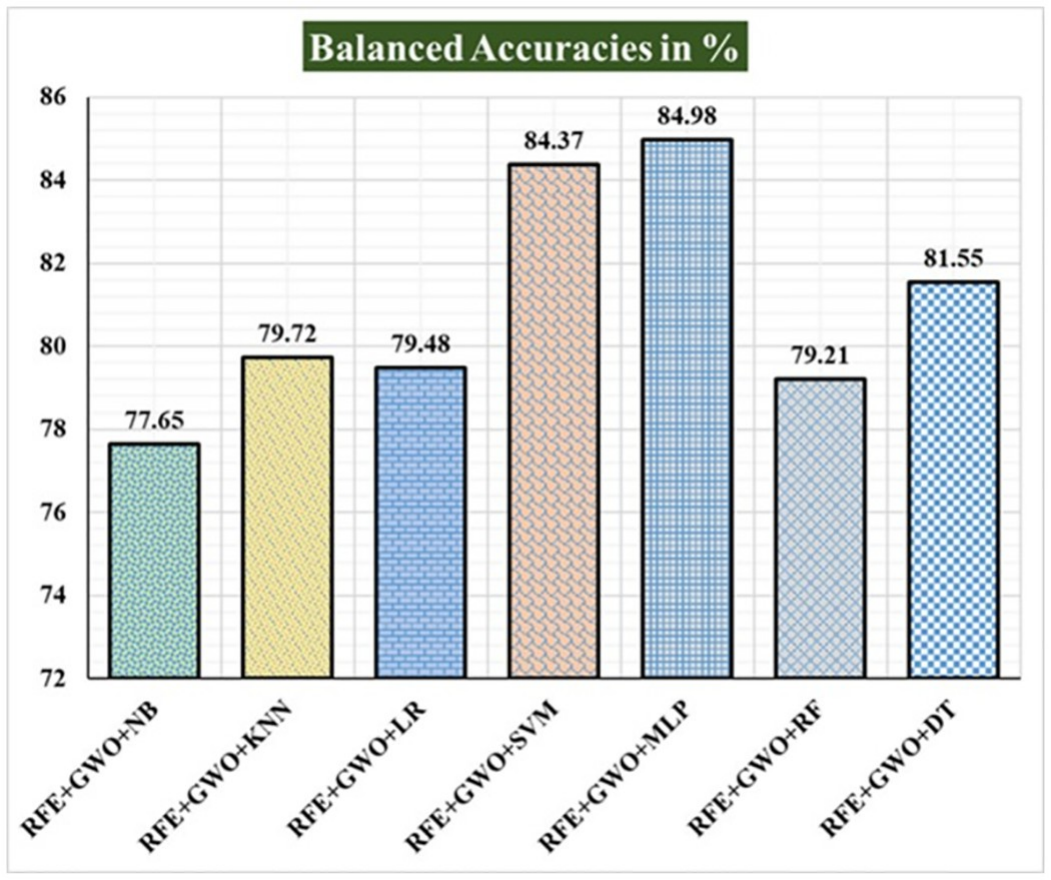
Recorded balanced accuracies in % for the hybrid ML approaches on the WPBC dataset.

**Fig 25:**
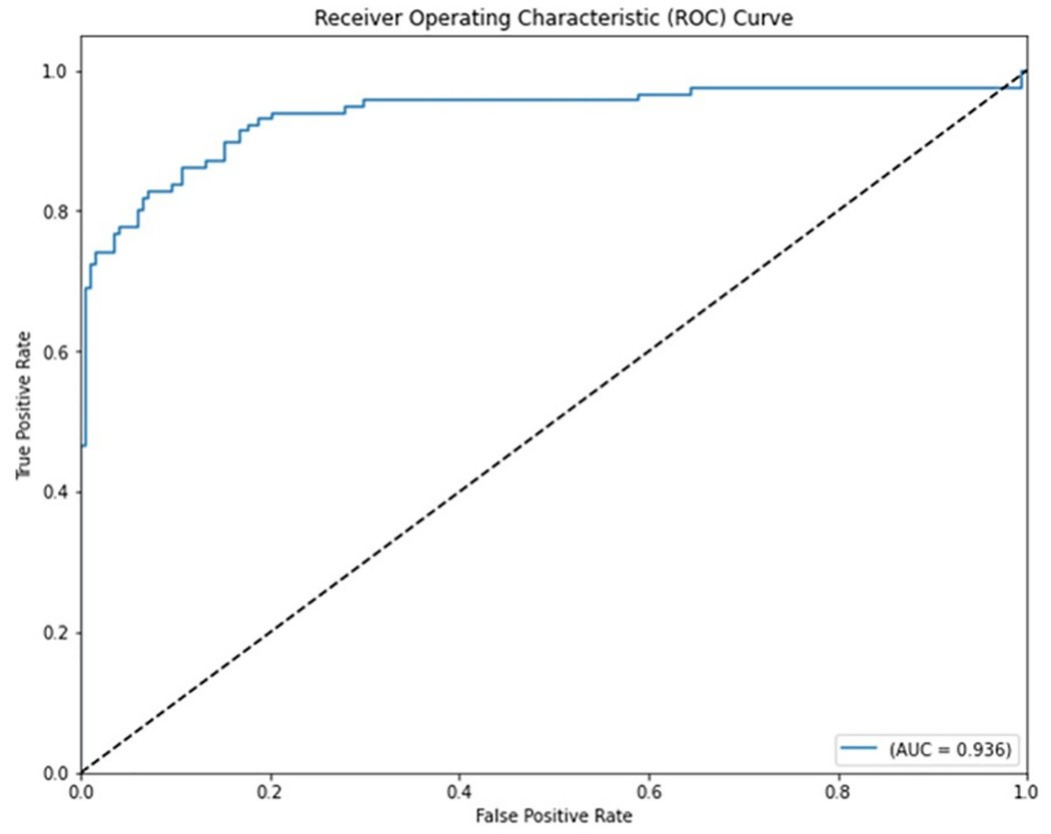
ROC curve with AUC value obtained employing the hybrid approach (RFE and GWO along with MLP) on WPBC dataset.

Seven traditional ML methods (NB, KNN, LR, SVM, MLP, RF, and DT) are combined with the feature selection method RFE and optimization technique GWO to generate the various ML-based hybrid approaches addressed in this paper. The second part of this research involved using these hybrid methods on the WPBC data set. In Table 4, we present the findings of in-depth studies of the effectiveness of the proposed ML-based hybrid techniques. Results for A_C_, M_R_, P_R_, S_N_, S_P_, F_S_, F_NR_, F_PR_, M_CC_, and B_A_ are displayed graphically in Figs 15–24. With an accuracy of 93.27%, precision of 95.56%, sensitivity of 96.63%, specificity of 73.33%, F1-score of 96.09%, etc., the "RFE+GWO+MLP" hybrid approach is clearly superior to the other six suggested hybrid approaches. Furthermore, as shown in Table 4, this hybrid ML approach also provides improved outcomes relative to other evaluative parameters considered in this work, such as misclassification rate, FNR, FPR, MCC, and balanced accuracy. Fig 25 depicts the results of calculating the ROC Curve and the AUC value for this suggested hybrid technique. The significance of this proposed method on the WPBC dataset is supported by the AUC value of 0.936 obtained using the suggested hybrid approach.

**Table 4:** Observed results employing various hybrid ML approaches on the WPBC dataset.

Hybrid ML Approaches	A_C_ in %	M_R_ in %	P_R_ in %	S_N_ in %	F_S_ in %	S_P_ in %	F_NR_ in %	F_PR_ in %	M_CC_ in %	B_A_ in %
RFE+GWO+NB	88.46	11.54	92.05	94.19	93.11	61.11	5.81	38.89	57.98	77.65
RFE+GWO+KNN	84.62	15.38	93.75	87.21	90.36	72.22	12.79	27.78	53.36	79.72
RFE+GWO+LR	89.42	10.58	93.18	94.25	93.71	64.71	5.75	35.29	60.43	79.48
RFE+GWO+SVM	92.31	7.69	94.32	96.51	95.4	72.22	3.49	27.78	72.07	84.37
RFE+GWO+MLP	93.27	6.73	95.56	96.63	96.09	73.33	3.37	26.67	72.01	84.98
RFE+GWO+RF	85.58	14.42	91.36	90.24	90.8	68.18	9.76	31.82	57.49	79.21
RFE+GWO+DT	86.54	13.46	93.83	89.41	91.57	73.68	10.59	26.32	58.75	81.55

### Comparative analysis

In order to show the novelty and significance of the proposed ML-based hybrid approach, we have added a comparative analysis. Tables 5 and 6 display the comparison between the proposed ML-based hybrid approach and the considered relevant state-of-the-art works, based on WDBC and WPBC datasets respectively, in terms of the findings obtained for accuracy, precision, specificity, sensitivity, F1-score, and AUC. The proposed work is found to be superior to and inferior to others on several evaluation parameters in both of the datasets, WDBC and WPBC, as depicted in Tables 5 and 6. Although the proposed work slightly fails to outperform these similar existing works based on the WDBC dataset as depicted in Table 5, whereas, it outperforms similar existing works based on the WPBC dataset as depicted in Table 6.

**Table 5:** Comparative analysis of the proposed hybrid approach to the considered state-of-the-art works based on WDBC dataset.

Findings	References			
	[8]	[14]	[20]	Proposed Work
Accuracy (in %)	98.12	98.82	99.20	98.25
Precision (in %)	99.20	-	97.40	98.13
Sensitivity (in %)	97.85	98.41	97.40	99.06
Specificity (in %)	-	99.07	-	96.92
F1-Score (in %)	-	-	97.40	98.59
AUC	-	0.9994	0.995	0.982

**Table 6:** Comparative analysis of the proposed hybrid approach to the considered state-of-the-art works based on wpbc dataset.

Findings	References		
	[18]	[20]	Proposed Work
Accuracy (in %)	80.00	78.60	93.27
Precision (in %)	80.00	77.70	95.56
Sensitivity (in %)	62.00	77.20	96.63
Specificity (in %)	-	-	73.33
F1-Score (in %)	76.00	78.00	96.09
AUC	0.81	0.789	0.936

## Conclusion and future scope

The ML-based hybrid approach suggested here makes use of not one but two breast cancer datasets: WDBC and WPBC. This investigation used RFE and GWO to further analyse and clarify this raw data. Both datasets undergo preliminary data processing, including imputation, scaling, and other methods. Second, an RFE selects the most numerous and pertinent features from the training datasets in order to accurately forecast the target variable. The GWO determined that the most effective combination of the selected features was necessary for a precise response. Using an 80/20 split, we examined the effectiveness of the proposed method. Therefore, the proposed hybrid technique selected features and improved breast cancer and recurrence classification accuracy. Several studies have shown accuracies of 98.25% and 93.27% on the WDBC and WPBC datasets, respectively; precisions of 98.13% and 95.56%; sensitivities of 99.06% and 96.63%; specificities of 96.92% and 73.33%; F1-scores of 98.59% and 96.09%; and area under the curves (AUCs) of 0.982 and 0.936. The hybrid method’s superior feature selection enhanced the precision of performance indicators for breast cancer and recurrence classification.

The advantages of this study include the hybridization of RFE and GWO with the conventional ML approaches. The use of RFE enables the identification and selection of the most relevant features, contributing to improved model efficiency and interpretability. GWO optimization facilitates the fine-tuning of model parameters, enhancing the convergence and effectiveness of the machine learning algorithms. We have also compared and contrasted the proposed hybrid ML-based approach with some of the similar state-of-the-art works showing the novelty and significance of the study.

Every research has advantages and disadvantages. The use of multiple ML approaches and optimization techniques may lead to increased computational demands. While GWO optimization contributes to model refinement, the effectiveness of the hybrid model is dependent on the careful tuning of hyperparameters. Sensitivity to hyperparameter choices is a common limitation shared by optimization-based methods, and we highlight the need for thoughtful parameter selection. The results of this investigation can be improved by using the ensemble methods to more breast cancer and breast cancer recurrence datasets with unique characteristics.

## References

[1] Arnold M, Morgan E, Rumgay H, Mafra A, Singh D, Laversanne M, et al. Current and future burden of breast cancer: Global statistics for 2020 and 2040. The Breast. 2022 Dec 1;66:15–23. doi: 10.1016/j.breast.2022.08.010 36084384 PMC9465273

[2] Narod SA, Iqbal J, Miller AB. Why have breast cancer mortality rates declined?. Journal of Cancer Policy. 2015 Sep 1;5:8–17.

[3] Saxena S, Shukla S, Gyanchandani M. Breast cancer histopathology image classification using kernelized weighted extreme learning machine. International Journal of Imaging Systems and Technology. 2021 Mar;31(1):168–79.

[4] Pati A, Panigrahi A, Sahu B, Sahoo G, Dash M, Parhi M, et al. FOHC: Firefly Optimizer Enabled Hybrid approach for Cancer Classification. International Journal on Recent and Innovation Trends in Computing and Communication. 2023 Jul 13;11(7s):118–25.

[5] Pati A, Parhi M, Pattanayak BK, Singh D, Singh V, Kadry S, et al. Breast Cancer Diagnosis Based on IoT and Deep Transfer Learning Enabled by Fog Computing. Diagnostics. 2023 Jun 27;13(13):2191. doi: 10.3390/diagnostics13132191 37443585 PMC10340497

[6] Sahu B, Panigrahi A, Rout SK, Pati A. Hybrid multiple filter embedded political optimizer for feature selection. In2022 International Conference on Intelligent Controller and Computing for Smart Power (ICICCSP) 2022 Jul 21 (pp. 1–6). IEEE.

[7] Panigrahi A, Pati A, Sahu B, Das MN, Nayak DS, Sahoo G, et al. En-MinWhale: An ensemble approach based on MRMR and Whale optimization for Cancer diagnosis. IEEE Access. 2023 Sep 22.

[8] Gupta M, Gupta B. A comparative study of breast cancer diagnosis using supervised machine learning techniques. In2018 second international conference on computing methodologies and communication (ICCMC) 2018 Feb 15 (pp. 997–1002). IEEE.

[9] Jafarpisheh N, Nafisi N, Teshnehlab M. Breast cancer relapse prognosis by classic and modern structures of machine learning algorithms. In2018 6th Iranian Joint Congress on Fuzzy and Intelligent Systems (CFIS) 2018 Feb 28 (pp. 120–122). IEEE.

[10] Ferroni P, Zanzotto FM, Riondino S, Scarpato N, Guadagni F, Roselli M. Breast cancer prognosis using a machine learning approach. Cancers. 2019 Mar 7;11(3):328. doi: 10.3390/cancers11030328 30866535 PMC6468737

[11] Bayrak EA, Kırcı P, Ensari T. Comparison of machine learning methods for breast cancer diagnosis. In2019 Scientific meeting on electrical-electronics & biomedical engineering and computer science (EBBT) 2019 Apr 24 (pp. 1–3). Ieee.

[12] Naveen, Sharma RK, Nair AR. Efficient breast cancer prediction using ensemble machine learning models. In2019 4th International conference on recent trends on electronics, information, communication & technology (RTEICT) 2019 May 17 (pp. 100–104). IEEE.

[13] Zeng Z, Yao L, Roy A, Li X, Espino S, Clare SE, et al. Identifying breast cancer distant recurrences from electronic health records using machine learning. Journal of healthcare informatics research. 2019 Sep 15;3:283–99. doi: 10.1007/s41666-019-00046-3 33225204 PMC7678240

[14] Omondiagbe DA, Veeramani S, Sidhu AS. Machine learning classification techniques for breast cancer diagnosis. InIOP Conference Series: Materials Science and Engineering 2019 Jun 7 (Vol. 495, p. 012033). IOP Publishing.

[15] Shravya C, Pravalika K, Subhani S. Prediction of breast cancer using supervised machine learning techniques. International Journal of Innovative Technology and Exploring Engineering (IJITEE). 2019 Apr;8(6):1106–10.

[16] Gu D, Su K, Zhao H. A case-based ensemble learning system for explainable breast cancer recurrence prediction. Artificial Intelligence in Medicine. 2020 Jul 1;107:101858. doi: 10.1016/j.artmed.2020.101858 32828461

[17] Lou SJ, Hou MF, Chang HT, Chiu CC, Lee HH, Yeh SC, et al. Machine learning algorithms to predict recurrence within 10 years after breast cancer surgery: A prospective cohort study. Cancers. 2020 Dec 17;12(12):3817. doi: 10.3390/cancers12123817 33348826 PMC7765963

[18] Magboo VP, Magboo MS. Machine learning classifiers on breast cancer recurrences. Procedia Computer Science. 2021 Jan 1;192:2742–52.

[19] Alzu’bi A, Najadat H, Doulat W, Al-Shari O, Zhou L. Predicting the recurrence of breast cancer using machine learning algorithms. Multimedia Tools and Applications. 2021 Apr;80:13787–800.

[20] Zeid MA, El-Bahnasy KH, Abu-Youssef SE. An efficient optimized framework for analyzing the performance of breast cancer using machine learning algorithms. J. Theor. Appl. Inf. Technol.. 2022 Jul 31;100(14):5165–78.

[21] Ebrahim M, Sedky AA, Mesbah S. Accuracy Assessment of Machine Learning Algorithms Used to Predict Breast Cancer. Data. 2023 Feb 2;8(2):35.

[22] “Breast Cancer Wisconsin (Diagnostic) Data Set” archive.ics.uci.edu. https://archive.ics.uci.edu/dataset/17/breast+cancer+wisconsin+diagnostic (Accessed Jan. 27, 2023).

[23] “Breast Cancer Wisconsin (Prognostic) Data Set,” archive.ics.uci.edu. https://archive.ics.uci.edu/dataset/16/breast+cancer+wisconsin+prognostic (Accessed Mar. 7, 2023).

[24] Chi CL, Street WN, Wolberg WH. Application of artificial neural network-based survival analysis on two breast cancer datasets. InAMIA annual symposium proceedings 2007 (Vol. 2007, p. 130). American Medical Informatics Association. 18693812 PMC2813661

[25] Wolberg WH, Street WN, Heisey DM, Mangasarian OL. Computerized breast cancer diagnosis and prognosis from fine-needle aspirates. Archives of Surgery. 1995 May 1;130(5):511–6. doi: 10.1001/archsurg.1995.01430050061010 7748089

[26] Fathima MD, Samuel SJ, Raja SP. HDDSS: An Enhanced Heart Disease Decision Support System Using RFE-ABGNB Algorithm. International Journal of Interactive Multimedia & Artificial Intelligence. 2023 Jun 1(527).

[27] Mirjalili S, Mirjalili SM, Lewis A. Grey wolf optimizer. Advances in engineering software. 2014 Mar 1;69:46–61.

[28] Al-Tashi Q, Md Rais H, Abdulkadir SJ, Mirjalili S, Alhussian H. A review of grey wolf optimizer-based feature selection methods for classification. Evolutionary Machine Learning Techniques: Algorithms and Applications. 2020:273–86.

[29] Mirjalili S, Aljarah I, Mafarja M, Heidari AA, Faris H. Grey wolf optimizer: theory, literature review, and application in computational fluid dynamics problems. Nature-inspired optimizers: Theories, literature reviews and applications. 2020:87–105.

[30] Pati A, Panigrahi A, Nayak DS, Sahoo G, Singh D. Predicting pediatric appendicitis using ensemble learning techniques. Procedia Computer Science. 2023 Jan 1;218:1166–75.

[31] Rout SK, Sahu B, Panigrahi A, Nayak B, Pati A. Early Detection of Sepsis Using LSTM Neural Network with Electronic Health Record. InAmbient Intelligence in Health Care: Proceedings of ICAIHC 2022 2022 Nov 23 (pp. 201–207). Singapore: Springer Nature Singapore.

[32] Pati A, Parhi M, Pattanayak BK. A review on prediction of diabetes using machine learning and data mining classification techniques. International Journal of Biomedical Engineering and Technology. 2023;41(1):83–109.

[33] Pati A, Parhi M, Pattanayak BK. IHDPM: An integrated heart disease prediction model for heart disease prediction. International Journal of Medical Engineering and Informatics. 2022;14(6):564–77.

[34] Nayak DSK, Pati A, Panigrahi A, Sahoo S, Swarnkar T. ReCuRandom: A hybrid machine learning model for significant gene identification. InAIP Conference Proceedings 2023 Jun 8 (Vol. 2819, No. 1). AIP Publishing.

[35] Pati A, Parhi M, Pattanayak BK, Singh D, Samanta D, Banerjee A, et al. Diagnose Diabetic Mellitus Illness Based on IoT Smart Architecture. Wireless Communications and Mobile Computing. 2022 Aug 25;2022.

[36] Tripathy J, Dash R, Pattanayak BK, Mishra SK, Mishra TK, Puthal D. Combination of reduction detection using TOPSIS for gene expression data analysis. Big Data and Cognitive Computing. 2022 Feb 23;6(1):24.

[37] Stark GF, Hart GR, Nartowt BJ, Deng J. Predicting breast cancer risk using personal health data and machine learning models. Plos one. 2019 Dec 27;14(12):e0226765. doi: 10.1371/journal.pone.0226765 31881042 PMC6934281

